# Transcriptome and genome-wide analysis of the potential role of *SKP1* gene family in the development of floral organs of two related species of *Allium fistulosum*

**DOI:** 10.3389/fpls.2024.1470780

**Published:** 2024-11-07

**Authors:** Ning Wang, Chenyi Lin, Zhongmin Yang, Dan Zhao

**Affiliations:** College of Horticulture, Xinjiang Agricultural University, Urumqi, Xinjiang, China

**Keywords:** *A. fistulosum* var. *viviparum* Makino, *A. galanthuma*, anther development, transcriptome, AfSKP1 gene family, expression analysis

## Abstract

*Allium fistulosum* is an important plant germplasm resource, rich in nutrients and possessing unique medicinal value. However, due to its small floral organs, low seed setting rate of a single flower, high cost of artificial emasculation, and artificial pollination, the use of male sterile lines to prepare *Allium* hybrids has become a common choice. In this study, *A. fistulosum* var. *viviparum* Makino and *A. galanthum* were used as materials to study the regulation mechanism of anther development, aiming to provide a reference for male sterility. Through transcriptome differential gene screening and genome-wide bioinformatics analysis, 34 *SKP1* (S-phase kinase-associated protein 1) genes (*AfSKP1-1* to *AfSKP1-34*) were identified in the whole genome of *A. fistulosum.* The *AfSKP1* genes are unevenly distributed on eight chromosomes. Furthermore, two pairs of collinear relationships are evident among family members, and fragment replication events between *AfSKP1* genes have been identified. The phylogenetic tree analysis demonstrated that the *AfSKP1, AtSKP1, OsSKP1*, and *SlSKP1* genes were clustered into six groups, exhibiting a gene structure analogous to that observed in members of an evolutionary classification. A combination of gene structure and phylogenetic analysis revealed the presence of cis-acting elements associated with growth, hormone regulation, and stress response within the *AfSKP1* genes. Furthermore, expression analysis demonstrated that the *AfSKP1* genes exhibited differential expression patterns across various tissues of *A. fistulosum*. The tissue-specific expression of the *AfSKP1* gene was verified by Real-Time PCR. A comparison of the two materials revealed significant differences in the expression of the *AfSKP1-8* gene in floral buds, the *AfSKP1-11* gene in inflorescence meristems, and the *AfSKP1-14* gene in inflorescence meristems, scapes, and floral buds. The results indicated that the three genes may be involved in anther development, thereby providing a theoretical basis for further study of floral organ development and pollen development in *AfSKP1* family members.

## Introduction

*Allium fistulosum* is a valuable plant germplasm resource that has served as a popular condiment in culinary dishes since ancient times, offering a wide range of nutritional benefits. It is not only rich in carbohydrates and proteins but also contains significant amounts of calcium, iron, phosphorus, and various vitamins (Michael, 2021). The conventional variety, which results from natural pollination, typically exhibits low yield and poor quality. In contrast, F1 hybrids demonstrate considerable heterosis, often increasing yield by 20% to 50% while significantly enhancing resistance and overall quality. However, challenges such as small floral organs, a low seed-setting rate per flower, and the high costs associated with artificial emasculation and pollination have led breeders to favor the use of male sterile lines for hybridization in *Allium* plants. By employing male sterile materials that do not produce viable pollen, the need for artificial emasculation is eliminated, thereby reducing labor costs and expediting the breeding and seed production processes. As closely related species, *A. fistulosum* var. *viviparum* Makino and *A. galanthum* exhibit strong drought and cold tolerance, as well as remarkable adaptability to both low and high temperatures in their environment. These traits can provide valuable genetic resources for enhancing the stress resistance of other Allium plants (Michael, 2021; Yamashita et al., 2000). Additionally, as a naturally male-sterile material, the genetic resources contained in *A. fistulosum* var. *viviparum* Makino require further development and utilization, and its sterility mechanism warrants additional investigation.

Male sterility is primarily characterized by the absence of stamens, the presence of abnormal stamens, irregular anther development, pollen deformities, inactivity, and abnormal germination in bisexual plants. Research on anther dehiscence has revealed that multiple genes and molecular mechanisms are integral to this process. In *Arabidopsis thaliana*, *AtMYB26* has been identified as a crucial regulator that directly induces the expression of *NST1* and *NST2*. These two genes are involved in the formation of secondary thickening in anthers, ensuring the specificity of secondary thickening sites and thereby facilitating anther opening (Yang et al., 2017). In *Gossypium hirsutum*, the interaction between *GhMYB24* and *GhJAZ1* has been shown to influence stamen development, leading to anther dehiscence and a reduction in pollen grain production, which underscores the significant role of *GhMYB24* in both anther dehiscence and pollen development (Li et al., 2013). Additionally, the *NAC* transcription factor family is also critical in the anther development of *A. thaliana*. These factors promote the thickening of the secondary wall in the endodermis cells of the anther by regulating lignin synthesis, thus modulating anther dehiscence (Mitsuda et al., 2007). SKP1 (S-phase kinase-associated-protein 1) has been reported in different crops and is mainly involved in several biological processes including anther and pollen development (Hellmann and Estelle, 2002; Hong et al., 2013; Francesca et al., 2016).

SKP1 as an essential backbone protein in the SCF complex, binds to both Cul1 and F-box proteins. Studies indicate that *SKP1* serves as a crucial backbone protein within the SCF complex, facilitating interactions with both Cullin1 and F-box proteins. It plays a significant role in numerous physiological growth and developmental processes in plants, including those regulated by growth hormones, gibberellins, meiosis, jasmonic acid, and ethylene (Xu et al., 2010; Liu et al., 2015; Wang et al., 2015). The *ask-1* mutant, derived from the mutagenesis of *A. thaliana ASK1* using the AC/Ds transposon, exhibits male sterility (Sundaresan et al., 1995). This male sterility arises from the involvement of the *ASK1* gene in male gametogenesis, where *ask1* mutants demonstrate abnormal separation of meiotic homologous chromosomes in male gametes. This abnormality leads to irregular meiosis in the pollen mother cell and, consequently, to the improper development of male gametes, resulting in male sterility (Yang et al., 1999; Wang and Yang, 2006). In *Arabidopsis*, AtSKP1-19 interacts with SAF1 to thicken the secondary wall of the inner wall of the anther, resulting in abnormal anther dehiscence (Kim et al., 2011). Furthermore, meiosis-associated *SKP1-like* genes have been identified in *Oryza sativa*, *Hordeum vulgare*, and *Triticum aestivum* (Hoopen et al., 2000; Sasaki et al., 2003; Hong et al., 2013; Senda et al., 2013). The down-regulation of the *SKP1* gene during the development of pollen grains in *T. aestivum* adversely affects the ubiquitination degradation pathway of proteins, which in turn leads to male sterility (Wang et al., 2015). Additionally, *SKP1-like* genes *LSK1-LSK3*, which influence the development of pollen and pollen tubes, have been identified in *Lilium species* (Chang et al., 2009). Although there is a relative paucity of studies focusing on *SKP1* genes in *Allium* species, the functional insights gained from *SKP1* genes in other plant species suggest that they may also play a critical role in the growth and development of *Allium* species, as well as in the mechanisms underlying male sterility. Therefore, investigating the *SKP1* gene in *Allium* species may elucidate its mechanisms of action in these processes and provide novel molecular tools for crop improvement.

In view of this, this study used paraffin section method and MTT staining method to analyze the reasons for the abortion of *A. fistulosum* var. *viviparum* Makino, and then identified the *AfSKP1* gene family by transcriptome sequencing combined with bioinformatics methods. The structure, conserved domain and tissue-specific expression of these genes were studied, and the characteristics of *AfSKP1* gene family members were analyzed from the aspects of phylogenetic evolution, chromosome distribution, protein physicochemical properties, and Motif prediction. The study also examined the cis-acting elements, expression patterns, and protein interaction networks of the gene promoters of family members. Furthermore, the expression patterns in inflorescence meristems, floral buds, scapes, and leaves were verified by qRT-PCR. This research provides a theoretical basis for analyzing the mechanism of the *AfSKP1* family’s involvement in the sterility of *A. fistulosum* var. *viviparum* Makino. It also offers a foundation for further studying the floral organ development and pollen development of *AfSKP1* family members.

## Materials and methods

### Plant materials and treatments

The materials used in this study were *A. galanthum* and *A. fisulosum* var. *viviparum* Makinot, which were obtained from the germplasm resource nursery of Sanping Base at Xinjiang Agricultural University. Anthers at different developmental stages were collected and preserved in FAA fixative (37% formaldehyde 10 mL, anhydrous ethanol 50 mL, glacial acetic acid 5 mL, ddH_2_O 35 mL). Leaves, scapes, inflorescence meristems, and floral buds were also collected. Three replications were conducted for each treatment, and the collected materials were snap-frozen with liquid nitrogen and stored in a refrigerator at -80°C.

### Paraffin sections observation of anthers

According to the classification period of anthers in *A. thaliana* (Sanders et al., 1999), the anthers at the developmental stages of S12-S14 (pollen maturation-anther dehiscence pollen release) were taken and stored in FAA fixative (37% formaldehyde 10 mL, anhydrous ethanol 50 mL, glacial acetic acid 5 mL, ddH_2_O 35 mL). After gradient dehydration and paraffin embedding, the fixed sample material was sliced using a slicer with a thickness of 6 μm. The excess wax on the surface was washed off, and then the sample was dyed with safranine green dye, followed by dyeing with neutral gum sealant. The slices were dried before micro observation and imaging.

### Detection of pollen activity and stigma pollinability

The pollen viability and stigma receptivity were determined using the methyl thiazolyl tetrazolium (MTT) staining method. A MTT sucrose solution (100 mg MTT dissolved in 5 mL of 50 g/L sucrose solution) was prepared, and the stigma was fully immersed in the dye solution. After 15 minutes at room temperature in the dark, the changes in the stigma were observed under an anatomical microscope, and the color changes of the stigma were noted post-treatment. Another MTT solution was prepared (500 mg of MTT dissolved in 100 mL of distilled water). The anthers that were about to burst were dissected to create a pollen suspension, and the solution was added drop by drop to a concave slide. After the addition of the MTT solution, the cover glass was placed over it and the slide was kept in a thermostat at 25°C in the dark for 15 minutes and observed under a microscope. Healthy pollen grains were stained blue-purple or reddish-brown, while inactive pollen grains were stained lightly or colorless (Dafni, 1992).

### Transcriptome sequencing

High throughput sequencing (Hlumina Novaseq 6000) was contracted through Novaseq Biotechnology Co. Samples were sequenced with 3 replicates for each sample and 17 gene libraries. It is noteworthy that three replicates from the flower buds of *A. galanthum* were contaminated with one replicate; however, all data originated from the same batch and this contamination did not compromise the integrity of the data. The raw data generated by the high-throughput sequencer were subjected to a cleaning process that involved the removal of low-quality sequences, spliced sequences, and sequences containing ambiguous bases. The cleaned data were subsequently aligned to the *A. fistulosum* reference genome using HISAT2 and StringTie software for sequence comparison and transcript assembly (Ewing et al., 1998; Kim et al., 2015; Pertea et al., 2015). The genomic data for A. fistulosum were obtained from the China National Sequence Archive (CNSA) at the following URL: https://ftp.cngb.org/pub/CNSA/data5/CNP0002276/CNS0461863/CNA0047403/chr.Afis.genome.fa.gz.

### Differential expression analysis

The number of corresponding reads for each transcript was quantified, and gene expression was measured using FPKM (fragments per kilobase of transcript per million fragments mapped (FPKM) (Trapnell et al., 2010). Differential gene expression analysis was conducted utilizing DESeq2 (version 1.20.0), with genes that satisfied the criteria of a *P value* (padjust) < 0.05 and |log2(fold change)| > 1 classified as differentially expressed genes (DEGs) (Love et al., 2014). Additionally, correlation analysis, principal component analysis, Gene Ontology (GO), and Kyoto Encyclopedia of Genes and Genomes (KEGG) enrichment analyses were performed. The GO and KEGG enrichment analyses were executed using the Allium BD online software (https://allium.qau.edu.cn/Allium/tools_enrichment.php), with the Benjamini-Hochberg (BH) method employed for multiple test correction. A corrected *P value* (padjust) < 0.05 was deemed indicative of significant enrichment of genes associated with GO functions and KEGG pathways (Yang et al., 2024). Heat maps of RNA-seq data were generated using the HeatMap Illustrator tool within TBtools software.

### Identification of *AfSKP1* genes

In order to identify potential *SKP1* genes in *A. fistulosum*, genomic data for *A. fistulosum* was retrieved from the China National Species Archive (CNSA) (https://ftp.cngb.org/pub/CNSA/data5/CNP0002276/CNS0461863/CNA0047403/chr.Afis.genome.fa.gz). Additionally, genomic data for *Solanum lycopersicum* (SL3.0, GCA_000188115, http://plants.ensembl.org/), *A. thaliana* (TAIR10, GCA_000001735, http://plants.ensembl.org/), and *Oryza sativa* (IRGSP-1.0, GCA_001433935, http://plants.ensembl.org/) were obtained from the Ensembl Plants database (http://plants.ensembl.org/). Subsequently, the *SKP1* domain hidden Markov model (ID: PF01466) was downloaded from the Pfam database (http://pfam.xfam.org/) and the Hmmer tool was used to search and compare the *SKP1* genome-wide proteins of *A. fistulosum* (E-value ≤ 1e−5) (Prakash et al., 2017). The retrieved proteins were then validated using the Conserved Domain Database program, accessible via NCBI (https://www.ncbi.nlm.nih.gov/), and the Search program, available through the Pfam database (http://pfam.xfam.org/).

### Analysis of physicochemical properties of *AfSKP1* protein and prediction of subcellular localization

The physicochemical properties of the AfSKP1 protein were determined using the Expasy online software (https://web.expasy.org/tools/) (Gasteiger et al., 2003). Subcellular localization was predicted using the online tool WoLF PSORT (https://wolfpsort.hgc.jp/).

### Phylogenetic tree and gene structure analysis of the *AfSKP1* gene

The *SKP1* protein sequences of *A. fistulosum*, *S. lycopersicum*, *A. thaliana*, and *O. sativa* were aligned using Muscle in MEGA 7.0 software. Family phylogenetic trees were constructed and analyzed using the Neighbor-joining (NJ) method with a bootstrap value of 1,000. The bootstrap repetition value was set at 1,000. The evolutionary tree was enhanced using the online tool EVOLVIEW (https://www.evolgenius.info/evolview-v2/) (Kumar et al., 2018). A conserved motif analysis was performed using MEME (https://meme-suite.org/tools/meme/) (Bailey et al., 2009). Gene structure maps and protein structural domain distribution maps were generated using TBtools software (Chen et al., 2020).

### Chromosomal localization and synteny analysis of the *AfSKP1* gene

The chromosomal location information of the *AfSKP1* gene was mapped using TBtools software, which also enabled the identification of its location distribution. MCScanX was employed for the covariance analysis of the *AfSKP1* gene (Wang et al., 2012), while the Circos tool was utilized to generate chromosome covariance maps (Krzywinski et al., 2009).

### Prediction of cis-acting elements in promoter sequences of the *AfSKP1* gene

The 2000 bp sequence upstream of the *AfSKP1* gene was extracted using TBtools software. Cis-acting elements in the promoter region were predicted using the Plant Care online website (http://bioinformatics.psb.ugent.be/webtools/plantcare/html/) (Lescot et al., 2002). The HeatMap tool in TBtools software was then utilized to generate an expression heat map.

### Prediction of functional interacting networks of *AfSKP1* protein

Protein interaction relationships were predicted using the STRING online website (https://string-db.org/), and the interaction networks were visualized with Cytoscape software (Szklarczyk et al., 2018; Su et al., 2014).

### Quantitative fluorescence analysis

Total RNA was extracted using a Tengen Biochemical Technology (Beijing) Co. extraction kit. The RNA-prep Plant Kit (DP441) was employed for the isolation of RNA, while cDNA was obtained through reverse transcription using the PrimeScript™ RT reagent Kit (Perfect Real Time). The qRT-PCR reaction system (20 μL) comprised 10 μL of 2x TB Green qPCR Master Mix, 8.2 μL of ddH2O, 0.4 μL of each upstream and downstream specific primer, and 1 μL of cDNA template. The reaction system for quantitative real-time polymerase chain reaction (qRT-PCR) (20 μL) comprised 10 μL of 2x TB Green qPCR Master Mix, 8.2 μL of deionized water (ddH2O), 0.4 μL of each upstream and downstream specific primer, and 1 μL of cDNA template. The reaction procedure was conducted on a 7500Fast (96) real-time fluorescence quantitative PCR instrument. It commenced with pre-denaturation at 94°C for 120 s, followed by denaturation at 94°C for 5 s, annealing at 15 s, and extension at 55°C for 10 s. This was repeated for 45 cycles. The relative expression was calculated using three biological replicates and the 2-^ΔΔCT^ method, with onion Actin serving as the internal reference gene. The relative expression represents the ratio between the treatment and control groups (Yao et al., 2020). The fluorescent quantitative PCR primers were designed based on the conserved sequences of the *AfSKP1* gene family members (**Table 1**).

**Table 1:** Primers used for real-time fluorescence quantitative PCR.

Gene	Forward primer sequence (5’ → 3’)	Reverse primer sequence (5’ → 3’)
*AfSKP1-8*	TCGCCATGGAATCTCAAACG	ACTGTTGACGTTCGGAATCG
*AfSKP1-11*	AACGATTCGGCACATGATCG	AATCTTGCTGTCGACGTTGG
*AfSKP1-14*	ACGCAAAGAAGCATGTTGCC	ACAAAATCAGCGTCCCAAGC
*AfActin*	CGAGATAGTGCGTGACATAAAGG	CGGGCACCTGAACCTCTCT

## Results

### Analysis of paraffin sections of anthers

**Figure 1:**
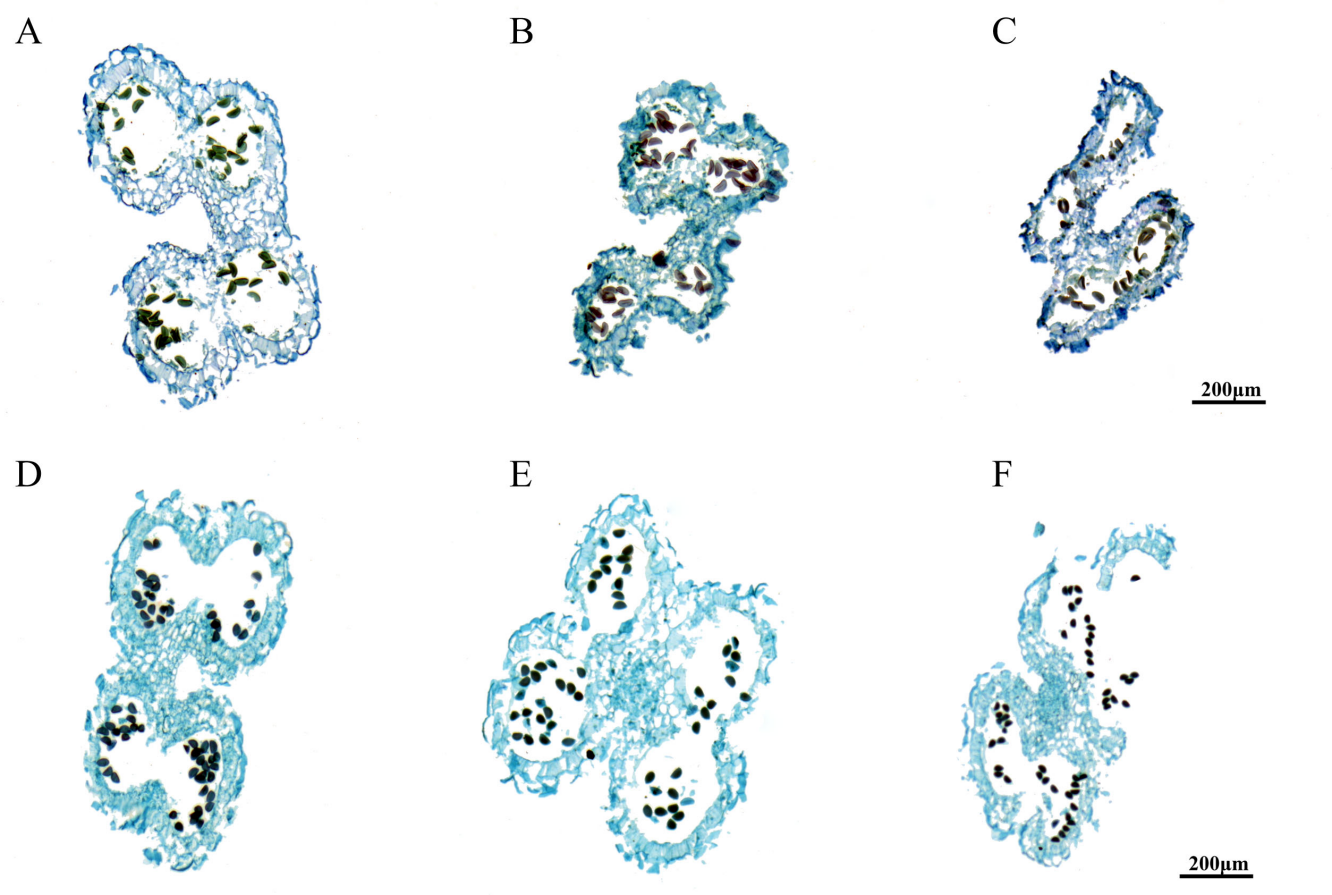
Paraffin sections of different anther development stages. **(A-C)** Shows anther development at stages S12-S14 of *Allium fisulosum* var. *viviparum* Makinot; **(D-F)** Shows anther development at stages S12-S14 of *Allium galanthum*.

The results of anther sectioning are illustrated in **Figure 1**. The anther structure of *A. fisulosum* var. *viviparum* Makinot exhibited a trend of shrinkage during the S12-S14 stages, which was in stark contrast to *A. galanthum*. At the S13 stage, the anther chamber could not crack, the maturation and release of pollen grains. Consequently, the anther became enveloped within itself by the S14 stage and ultimately detached along with the filaments. In contrast, at the S13 stage, the anther of *A. galanthum* was cracked, allowing for the release of mature pollen grains. By the S14 stage, the anther of *A. galanthum* had completed its function, collapsing to facilitate the successful release of pollen. At the mature stage of pollen grains (S13), the pollen grains of A. galanthum were full and deeply colored, while the pollen grains of *A. fisulosum* var. *viviparum* Makinot were deformed and lightly colored. *A. fisulosum* var. *viviparum* Makinot to develop mature pollen grains ultimately resulted in the abortion of its pollen.

### Analysis of pollen activity, stigmatic pollinability

**Figure 2:**
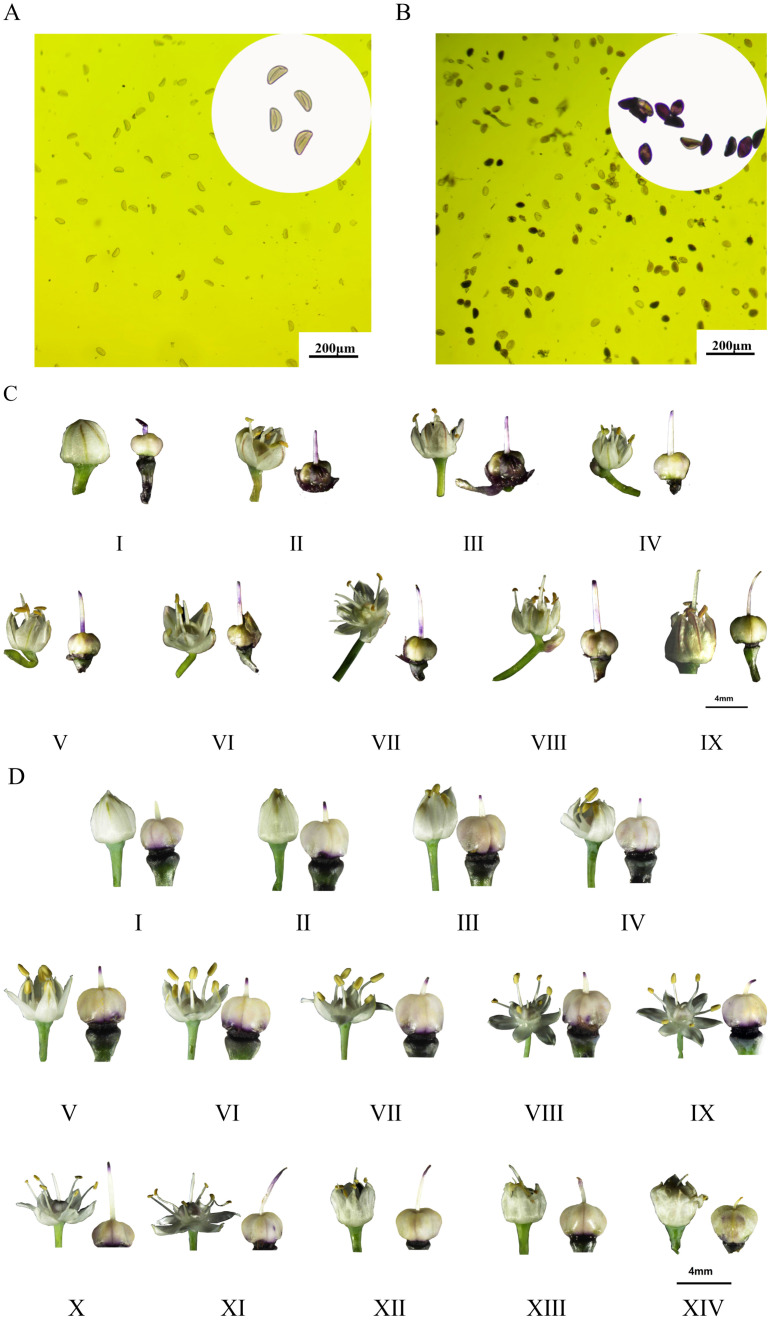
Determination of pollen viability, flowering dynamics and stigma receptivity. **(A, C)** Is for *Allium fisulosum* var. *viviparum* Makino; **(B, D)** is for *Allium galanthum*. The flowering of *Allium* fistulosum was divided into 9 periods (I-IX), including: bullet period, one anther period, two anther periods, three anther periods, four anther periods, five anther periods, six anther periods, closing period, wilting period; the flowering of *Allium* galanthum is divided into 14 periods (I-XIV), including: bullet period, mouth opening period, vomiting period, one anther period, two anther periods, three anther periods, four anther periods, five anther periods, six anther periods, such as long-term, beyond period, closing period, wilting period, expansion period.

The results of pollen treated with MTT solution are shown in **Figure 2**. The *A. galanthum* pollen is stained grey or light grey to indicate inactivity (**Figure 2A**), and the *A. fisulosum* var. *viviparum* Makinot pollen is stained blue or light blue to indicate vitality (**Figure 2B**). Stigmas treated with MTT sucrose solution were stained blue-violet to indicate viable stigmas. A dark color indicates high viability, while a stigma with no change in color indicates that the stigma is not viable or has lost viability. The *A. fisulosum* var. *viviparum* Makinot is divided into 9 periods, from before the florets open to the drying of the florets. The stigma is blue-violet throughout the development period and is receptive (**Figure 2C**). The A. galanthum is divided into 14 periods from the time of floret dehiscence to the time of floret dehiscence; except for the pre-floret dehiscence and ovary expansion periods, all other developmental periods of the blue-purple stigma are fertile (**Figure 2D**). The *A. fisulosum* var. *viviparum Makinot* exhibits fertility during the pre-floret dehiscence stage when compared to *A. galanthum*. However, *A. fisulosum* var. *viviparum* Makinot is incapable of producing viable pollen and lacks an ovary expansion period. Due to the inability of *A. fisulosum* var. *viviparum* Makinot to mature and release pollen, the assessment of pollen activity, stigma pollination, and overall pollination is limited to the application of chemical reaction MTT staining. Future research will involve conducting pollen tube growth tests through distant hybridization to further investigate its fertility.

### Expression analysis of differentially expressed genes

In order to find the important genes controlling anther development, combined with the results of paraffin section observation, RNA-seq analysis was carried out on the inflorescence meristems (before bolting), floral buds (before flowering) and leaves (before flowering) of the two materials. A total of 17 RNA libraries were prepared and sequenced. The sequencing generated 109.96 G of raw data and 106.30 G of filtered clean data, with a Q30 value exceeding 91.51%. The data obtained was of exceptional quality, making it well-suited for subsequent analysis.

**Figure 3:**
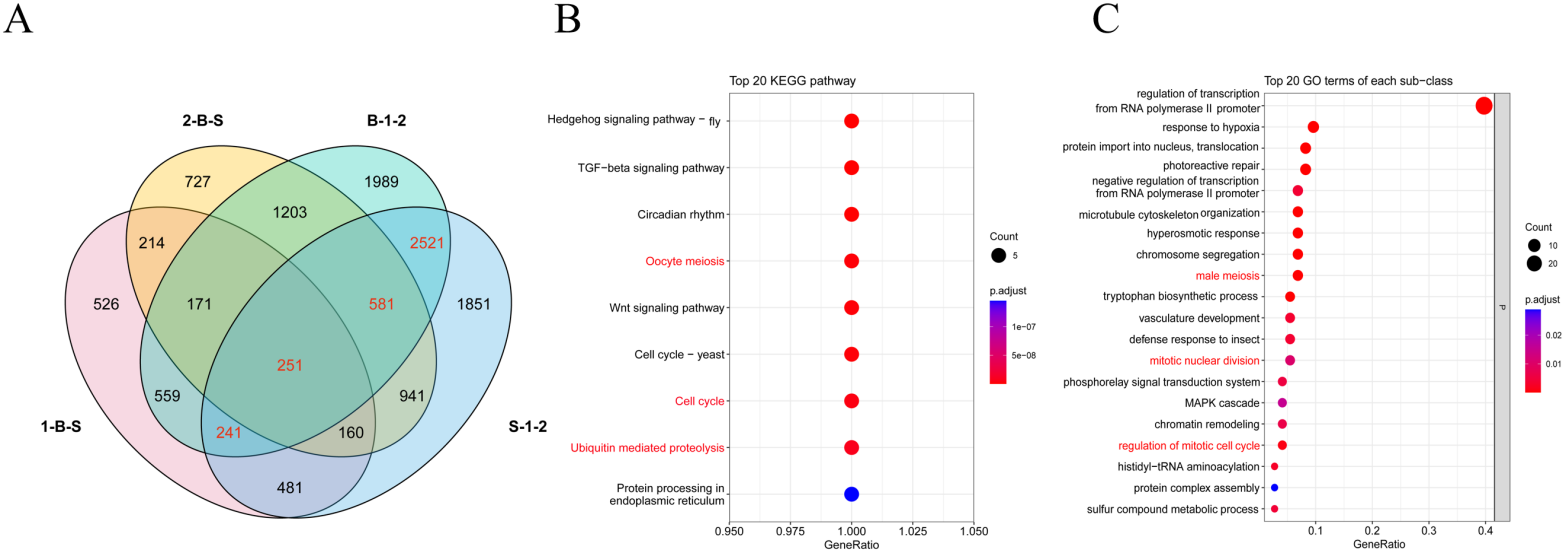
Analysis of the differentially expressed genes in four tissue site. **(A)** Shows the Venn diagram of DEGs. **(B, C)** Show the bubble plots of the screened DEGs enriched in KEGG and GO, respectively.

After quality control data processing, a total of 28,436 differentially expressed genes were identified by analyzing the differential expression of genes in different samples (**Figure 3A**). A total of 7072 DEGs were obtained from inflorescence meristems (S-1-2) of both materials, and 7516 DEGs were obtained from floral buds (B-1-2) of both materials. A total of 2603 DEGs were obtained from inflorescence meristems and floral buds of *A. fisulosum* var. *viviparum* Makinot (1-B-S). A total of 4,248 DEGs were obtained from inflorescence meristems and floral buds of *A. galanthum* (2-B-S). Combined with the previous anther phenotyping study, this investigation further examines the differential gene expression between two treatment groups, S-1-2 and B-1-2, with a specific focus on genes associated with floral organ development. In the S-1-2 treatment group, 3 *AfSKP1* genes—*AfSKP1-1*, *AfSKP1-2*, and *AfSKP1-8*—were identified, alongside 12 *MADS-box* genes and 38 *MYB* genes. Conversely, in the B-1-2 treatment group, the genes *AfSKP1-1*, AfSKP1-2, AfSKP1-8, AfSKP1-22, and AfSKP1-23 were detected. Collectively, these 5 *AfSKP1* genes, in addition to 14 *MADS-box* genes and 36 *MYB* genes, were catalogued (**Supplementary Table 1**). In the current study, functional analyses were conducted based on the three floral development-related differential genes identified in the preliminary overview. The results indicated that the functions of the *AfSKP1* genes are primarily associated with oocyte meiosis (KO04114), the cell cycle (KO04110), and ubiquitin-mediated proteolysis (KO04120) (**Figure 3B**). Additionally, these genes were linked to male meiosis (GO:0007140), mitotic nuclear division (GO:0007067), and the regulation of the mitotic cell cycle (GO:0007346) (**Figure 3C**). Consequently, it is hypothesized that the identification of the AfSKP1 gene may play a significant role in the regulation of anther development. Further investigation into the *AfSKP1* gene family is warranted to deepen our understanding of its functional implications.

### Characterization of *AfSKP1* gene family members

In this study, 34 potential *AfSKP1* genes were initially identified in *A. fisulosum* through genome-wide identification analysis and named *AfSKP1-1* to *AfSKP1-34* based on their positions in the genome chromosome. The results of protein physicochemical property analysis (**Table 2**) showed that the number of amino acid residues of *AfSKP1* family members ranged from 57 aa to 385 aa, and the molecular weights of the proteins ranged from 6556.28 to 43941.54. Among them, *AfSKP1-19* were the smallest with 55 aa and 6556.28, while *AfSKP1-9* were the largest with 385 aa and 43941.54. The isoelectric points ranged from 4.25 to 6.4, with *AfSKP1-18* being the lowest and *AfSKP1-13* the highest. All *AfSKP1* gene family proteins are hydrophilic except *AfSKP1-34* The secondary structures of proteins encoded by the *AfSKP1* gene family mainly include α-helix, β-sheet, and random coil. The 34 proteins encoded by the *AfSKP1* gene family were predominantly α-helix (42.58% - 86.21%) and random coil (10.94% - 45.38%), with less β-sheet (1.47% - 9.72%). Subcellular localization prediction (**Table 2**) indicated that 15 *AfSKP1* family members were localized in the cytosol, 8 in the mitochondria, 6 in the chloroplast, 4 in the nucleus, and only *AfSKP1-3* in the cytoskeleton.

**Table 2:** Physicochemical properties of amino acid sequences encoded by members of *AfSKP1* gene family.

Gene name	Gene ID	Chromosome location	Amino acid	Molecular weigh	Isoelectric point	Gravy	Subcellular localization	Protein secondary structure		
								a	b	c
*AfSKP1-1*	*AfisC1G02953*	chr1(545356150-545356845)	128	14792.75	4.75	-0.756	mitochondrial	66.41	3.91	28.12
*AfSKP1-2*	*AfisC1G02955*	chr1(545375479-545424103)	202	22574.7	5.22	-0.477	Chloroplast	46.53	4.95	38.61
*AfSKP1-3*	*AfisC1G07937*	chr1(1443549886-1443560745)	355	40626.72	5.29	-0.868	cytoskeleton	49.86	3.66	41.97
*AfSKP1-4*	*AfisC2G00216*	chr2(22968903-22971376)	164	18506.77	4.53	-0.549	nucleus	60.37	4.88	29.27
*AfSKP1-5*	*AfisC2G01572*	chr2(221598019-221598602)	64	7466.33	4.58	-0.602	cytosol	85.94	3.12	10.94
*AfSKP1-6*	*AfisC2G02657*	chr2(408420617-408424630)	165	18616.89	4.5	-0.49	cytosol	63.64	6.06	25.45
*AfSKP1-7*	*AfisC2G05170*	chr2(855109512-855127983)	164	18660.09	4.61	-0.505	mitochondrial	63.41	4.27	26.22
*AfSKP1-8*	*AfisC2G05173*	chr2(855632616-855643415)	164	18660.09	4.61	-0.505	mitochondrial	63.41	4.27	26.22
*AfSKP1-9*	*AfisC2G06197*	chr2(1012886451-1012902821)	385	43941.54	5.55	-0.814	mitochondrial	51.17	6.23	32.47
*AfSKP1-10*	*AfisC2G07235*	chr2(1162925631-1162926555)	157	17910.3	4.88	-0.445	nucleus	66.24	5.73	22.29
*AfSKP1-11*	*AfisC2G08961*	chr2(1478322550-1478335654)	164	18568.84	4.5	-0.576	cytosol	62.8	6.1	24.39
*AfSKP1-12*	*AfisC3G02524*	chr3(440368568-440370128)	171	19494.48	5.07	-0.011	mitochondrial	56.14	4.09	29.82
*AfSKP1-13*	*AfisC3G03026*	chr3(534728322-534773809)	357	40856.54	6.4	-0.796	Chloroplast	42.58	4.2	45.38
*AfSKP1-14*	*AfisC3G08204*	chr3(1458066615-1458068229)	159	17733	4.47	-0.384	cytosol	62.89	6.29	25.16
*AfSKP1-15*	*AfisC4G02565*	chr4(492628663-492641152)	146	16100.31	5.55	-0.325	cytosol	62.33	8.22	16.44
*AfSKP1-16*	*AfisC4G02592*	chr4(497580205-497583011)	72	8235.35	5.08	-0.357	Chloroplast	69.44	8.33	12.5
*AfSKP1-17*	*AfisC4G02593*	chr4(497761940-497777514)	146	16053.25	6.15	-0.307	Chloroplast	57.53	7.53	21.92
*AfSKP1-18*	*AfisC4G04467*	chr4(860213090-860214231)	58	6693.43	4.25	-0.333	cytosol	86.21	1.72	12.07
*AfSKP1-19*	*AfisC4G06122*	chr4(1128771848-1128772021)	57	6556.28	4.6	-0.416	cytosol	82.46	1.75	15.79
*AfSKP1-20*	*AfisC5G03987*	chr5(742711334-742712081)	159	18010.32	4.55	-0.378	Chloroplast	61.64	6.92	25.79
*AfSKP1-21*	*AfisC5G04629*	chr5(846339026-846339535)	68	7846.88	4.85	-0.357	mitochondrial	77.94	1.47	20.59
*AfSKP1-22*	*AfisC5G05165*	chr5(944794295-944795584)	155	17450.66	4.41	-0.448	cytosol	61.29	7.74	25.16
*AfSKP1-23*	*AfisC5G05934*	chr5(1092009485-1092012268)	164	18601.91	4.56	-0.524	mitochondrial	65.24	6.71	22.56
*AfSKP1-24*	*AfisC6G03278*	chr6(593553345-593553557)	70	8136.51	5.73	-0.314	cytosol	80	8.57	11.43
*AfSKP1-25*	*AfisC6G06107*	chr6(1078424272-1078425322)	167	19068.92	4.73	-0.136	cytosol	58.08	5.39	28.74
*AfSKP1-26*	*AfisC6G06201*	chr6(1093311224-1093311977)	159	18009.13	4.41	-0.405	mitochondrial	65.41	6.29	23.27
*AfSKP1-27*	*AfisC6G06203*	chr6(1093376204-1093376954)	159	17996.09	4.37	-0.389	cytosol	62.89	5.66	25.79
*AfSKP1-28*	*AfisC6G06204*	chr6(1093439850-1093440604)	159	17994.98	4.3	-0.369	cytosol	67.3	5.03	22.64
*AfSKP1-29*	*AfisC6G06205*	chr6(1093797412-1093798168)	160	18005.96	4.28	-0.363	cytosol	61.88	8.12	25
*AfSKP1-30*	*AfisC7G02706*	chr7(533032118-533033258)	58	6665.38	4.25	-0.374	cytosol	82.76	3.45	13.79
*AfSKP1-31*	*AfisC7G03759*	chr7(728368063-728368787)	159	17875.91	4.37	-0.354	cytosol	64.15	5.66	24.53
*AfSKP1-32*	*AfisC7G03760*	chr7(728374359-728374941)	100	11761.28	6.33	-0.816	nucleus	67	5	23
*AfSKP1-33*	*AfisC8G02609*	chr8(513391775-513392616)	176	19963.94	4.61	-0.172	nucleus	59.09	7.95	25
*AfSKP1-34*	*AfisC8G02613*	chr8(514217105-514217400)	72	8310.63	4.71	0.185	Chloroplast	63.89	9.72	11.11

a refers to α-helix, b refers to β-sheet, and c refers to random coil.

### Chromosomal localization of the *AfSKP1* gene family

**Figure 4:**
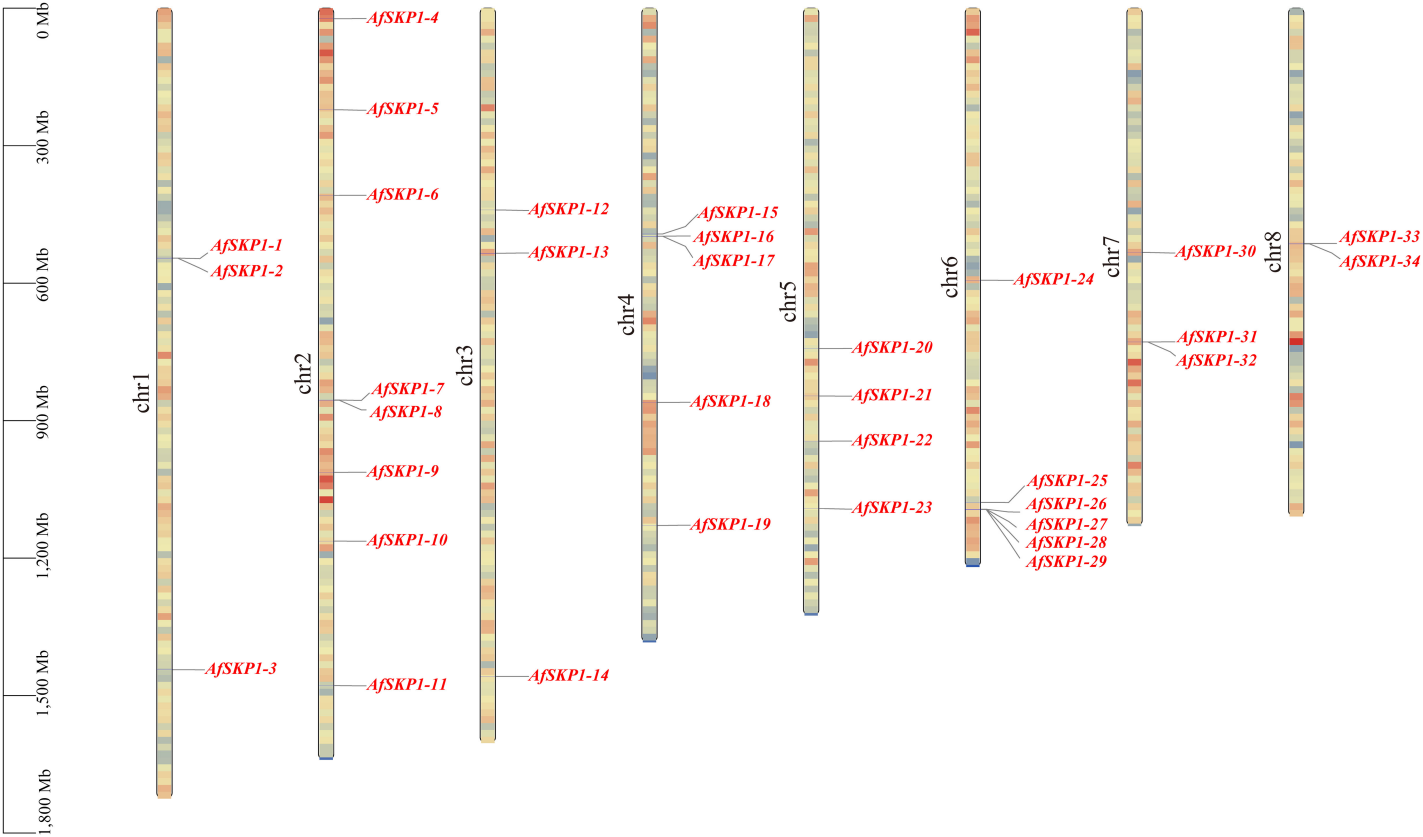
Chromosomal localization of *AfSKP1*gene. The different colors in the figure represent the density of the gene on the chromosome, which are represented by red yellow blue from high to low.

Chromosomal localization analysis showed (**Figure 4**) that 34 members of the *AfSKP1* gene family were distributed on 8 chromosomes. *AfSKP1* genes were mainly distributed on Chr2 (8), Chr4 (5), Chr5 (4), Chr6 (6), Chr1, Chr3, and Chr7 each had 3 members of the gene family distributed, and Chr8 had only 2 members of the gene family distributed. *AfSKP1* genes were distributed at both the upper and lower ends of chromosomes Chr1, Chr2, Chr3, and Chr4, and mainly at the lower ends of chromosomes Chr5, Chr6, and Chr7. The members of the *AfSKP1-15*~*AfSKP1-17* gene family are more concentrated at the upper end of the Chr4 chromosome, while the members of the *AfSKP1-25*~*AfSKP1-29* gene family are more concentrated at the lower end of the Chr6 chromosome. The formation of this distribution may be related to molecular events such as gene duplication, recombination and chromosomal remodeling.

### Phylogenetic classification of the *AfSKP1* gene

**Figure 5:**
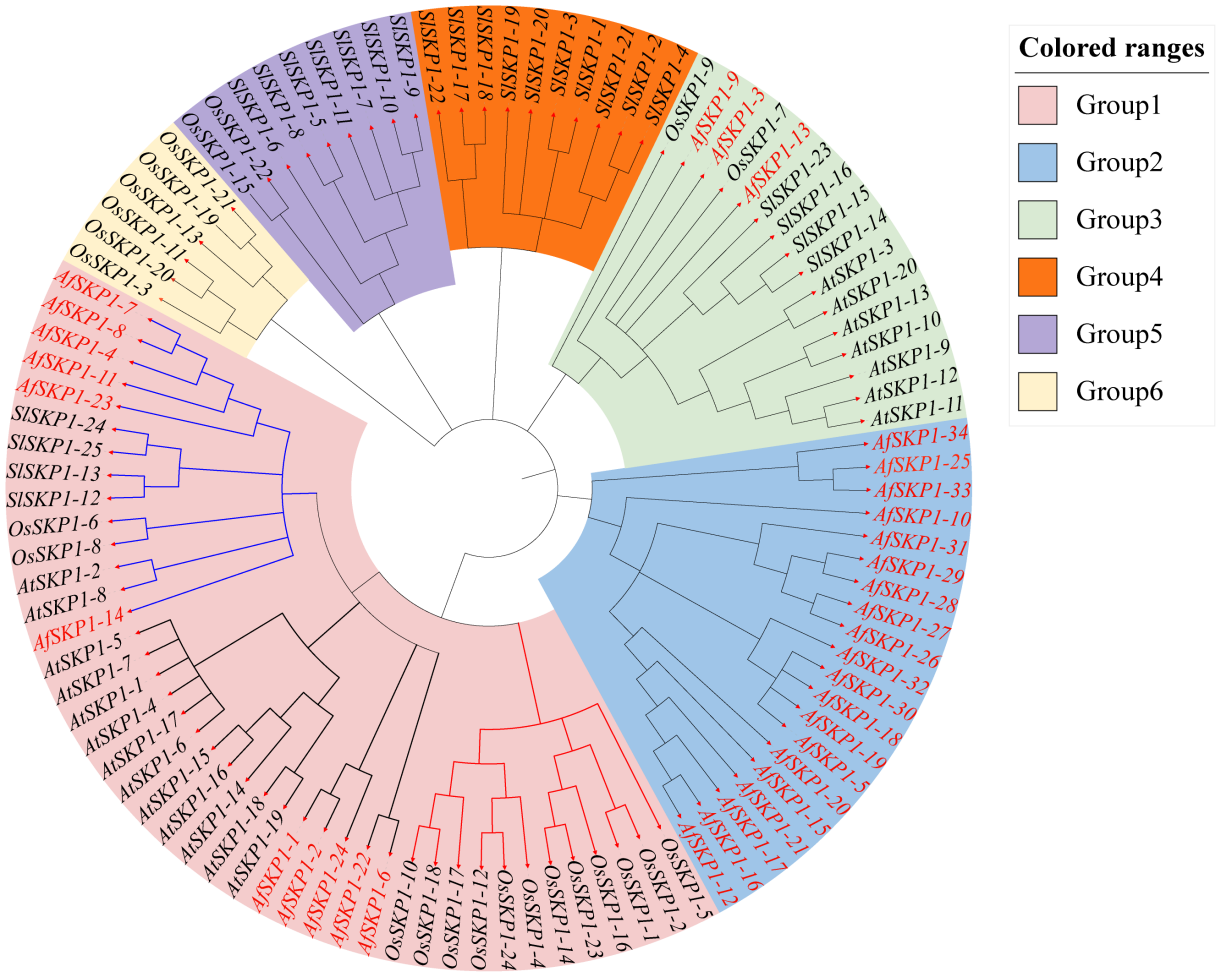
Phylogenetic analysis of *AfSKP1* genes in *Allium fisulosum* (*AfSKP1*), *Arabidopsis thaliana* (*AtSKP1*), *Oryza sativa* (*OsSKP1*), and *Solanum lycopersicum*(*SlSKP1*). The phylogenetic tree was constructed using the NJ method.

To clarify the phylogenetic relationship of *AfSKP1* genes, phylogenetic trees were constructed for the protein sequences of a total of 103 *SKP1* conserved structural domains of *A. fisulosum* (*AfSKP1*, 34), *A. thaliana* (*AtSKP1*, 20), *O. sativa* (*OsSKP1*, 24), and *S. lycopersicum* (*SlSKP1*, 25) (**Figure 5**). According to the protein sequence of *AfSKP1* family members, the phylogenetic tree was constructed, and the *SKP1* genes of these four species were divided into 6 groups. The *SKP1* gene family members in the same group had a similar genetic relationship. There were 42 *SKP1* genes in group I, including 11 *AfSKP1*, 13 *AtPIP5K*, 14 *OsSKP1*, and 4 *SlSKP1*; 20 *SKP1* genes in group II, all of which were *AfSKP1*; and 16 *SKP1* genes in group III, including 3 *AfSKP1*, 7 *AtSKP1*, 2 *OsSKP1*, and 4 *SlSKP1*. Group IV had a total of 10 *SKP1* genes, all of which were *SlSKP1*; group V had a total of 9 *SKP1* genes, containing 2 *OsSKP1* and 7 *SlSKP1*; and group VI had a total of 6 *SKP1* genes, all of which were *OsSKP1*. Meanwhile, group I was divided into two subgroups, containing six and five *AfSKP1* family members, respectively; genes within the same group usually have similar functional characteristics, and most of the *AfSKP1* genes were in groups I and II, thus suggesting that AfSKP1 gene may be involved in the regulation of floral organ development and pollen maturation.

### Analysis of gene structure and conserved motifs of *AfSKP1* gene family members

**Figure 6:**
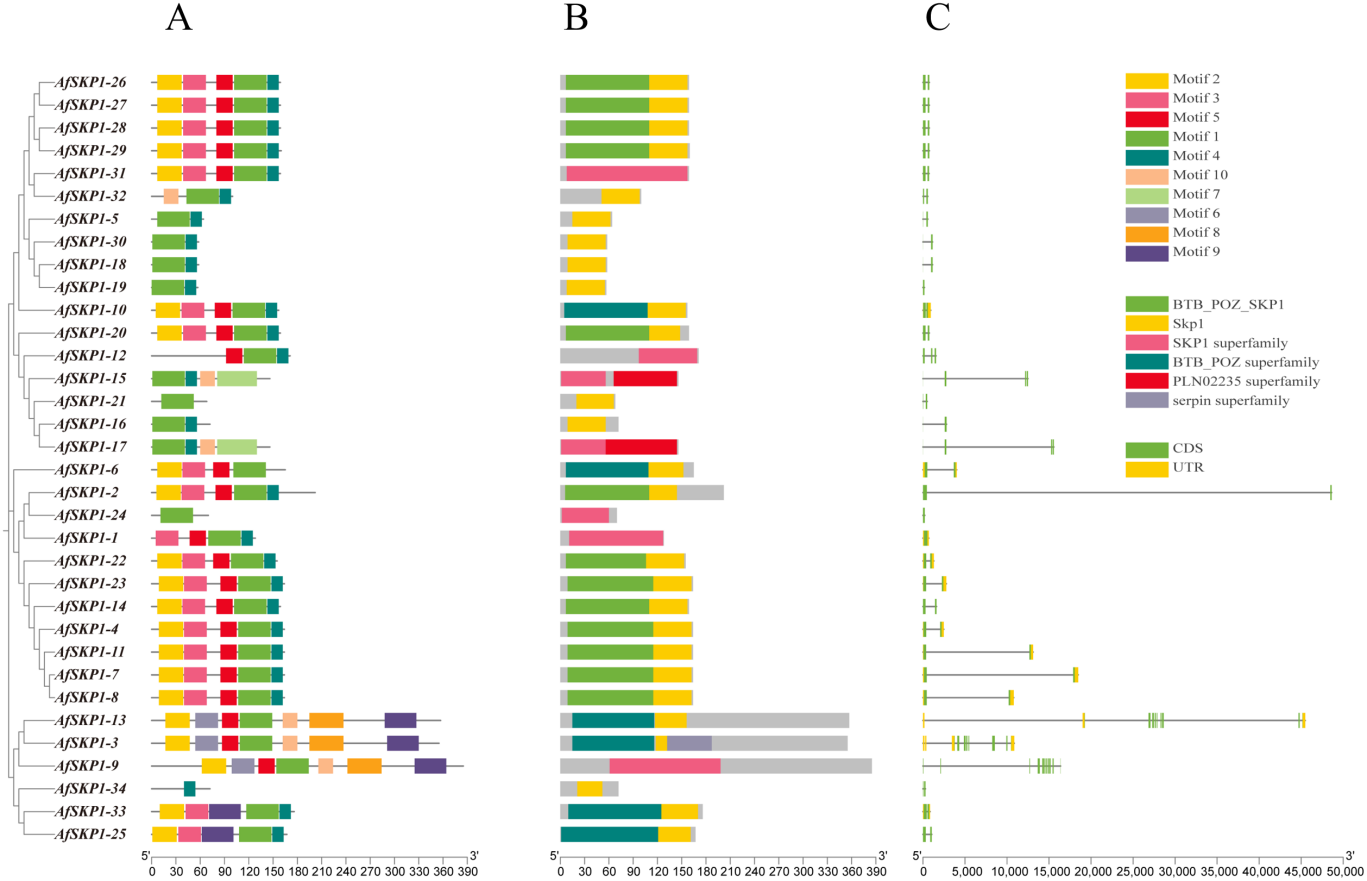
Gene structure diagram of *AfSKP1* gene family. **(A)** Conserved motifs in the family are depicted with different colors representing different motifs. A maximum of 9 motifs were searched with lengths between 10 and 50 residues. Motif occurrence was limited to zero or one occurrence per sequence. **(B)** Conserved domains found in family members are illustrated with different colors representing distinct conserved domains. **(C)** Gene structure of *AfSKP*1s is shown with yellow squares representing UTR, green squares representing CDS, and lines representing introns.

As shown in **Figure 6**, *AfSKP1* proteins exhibit various motif types. Among them, *AfSKP1-2*, *AfSKP1-5*, *AfSKP1-9*, *AfSKP1-12*, *AfSKP1-14* to *AfSKP1-21*, *AfSKP1-24* to *AfSKP1-32*, and *AfSKP1-34* contain only CDS (translated region), which may have implications for mRNA transport, stability, translational regulation, and incomplete gene annotation (Wu and Bazzini, 2023), other genes have both CDS (translated region) and UTR (untranslated region)(**Figure 6C**). Gene structure analysis revealed that the number of introns in the *AfSKP1* genome ranged from 1 to 11, with *AfSKP1-24* containing only 1 intron, *AfSKP1-9* containing 11 introns, and the remaining members of the *AfSKP1* gene family containing 2-7 introns. Introns are sequences that impede the linear expression of genes, increasing gene length and recombination frequency, and are advantageous for species evolution and gene expression regulation (Rose, 2018). The results indicated that the *AfSKP1-24* gene may have some function, but it did not significantly impact the evolution of *A*. fistulosum. *AfSKP1-9* may be more conducive to the evolution of *A*. fistulosum. Phylogenetic tree analysis revealed that gene family members in the same group share a relatively similar genetic relationship. For instance, *AfSKP1-26*, *AfSKP1-27*, *AfSKP1-28*, *AfSKP1-29*, and *AfSKP1-31* are clustered together and exhibit a close genetic relationship. Based on conserved motifs, the five *AfSKP1* genes in the same group have similar gene structures, indicating analogous functions. Similarly, *AfSKP1-2*, *AfSKP1-4*, *AfSKP1-7*, *AfSKP1-8*, *AfSKP1-11*, *AfSKP1-14*, *AfSKP1-22*, and *AfSKP1-23* are known to perform similar functions. Additionally, *34 AfSKP1* proteins were analyzed for 10 motifs, with 31 proteins sharing motif 1, suggesting its widespread distribution and high conservation in *AfSKP1* protein sequences. This indicates that motif 1 is widely distributed and strongly conserved in the AfSKP1 protein sequence, and that this structural domain is essential for the binding of SKP1 to Cullin family proteins, which is a key step in the formation of the SCF complex. (Zhao et al., 2003) Most *AfSKP1* members in the same subfamily also share conserved motifs, indicating functional conservation. *AfSKP1-34* exclusively contains motif 4 (Wu and Bazzini, 2023), suggesting a unique function, however, its specific role remains to be verified. (**Figure 6A**). Furthermore, specific motifs are present in distinct subfamilies, highlighting functional diversity in *AfSKP1* across different subfamilies.

The distribution of structural domains of the *AfSKP1* protein is depicted in **Figure 6B**. The results indicate that all *AfSKP1* members contain *SKP1* structural domains or SKP1 superfamily members. Specifically, 13 *SKP1* members include the BTB-POZ-SKP1 structural domain at the N-terminus. The BTB-POZ structural domain is a characteristic feature of *SKP1* structure, characterized by the presence of the BTB/POZ structural domain fold, which includes an α-helix cluster flanking a short β-sheet at the amino terminus. The BTB-POZ transcriptional regulator regulates inflorescence structure in tomato (Xu et al., 2016). This structure facilitates the ubiquitination of proteins involved in cell cycle progression, signaling, and transcription. Therefore, it is plausible that this function is also present in *A. fisulosum*, but further studies are necessary.

### Covariance analysis of *AfSKP1* gene family members

**Figure 7:**
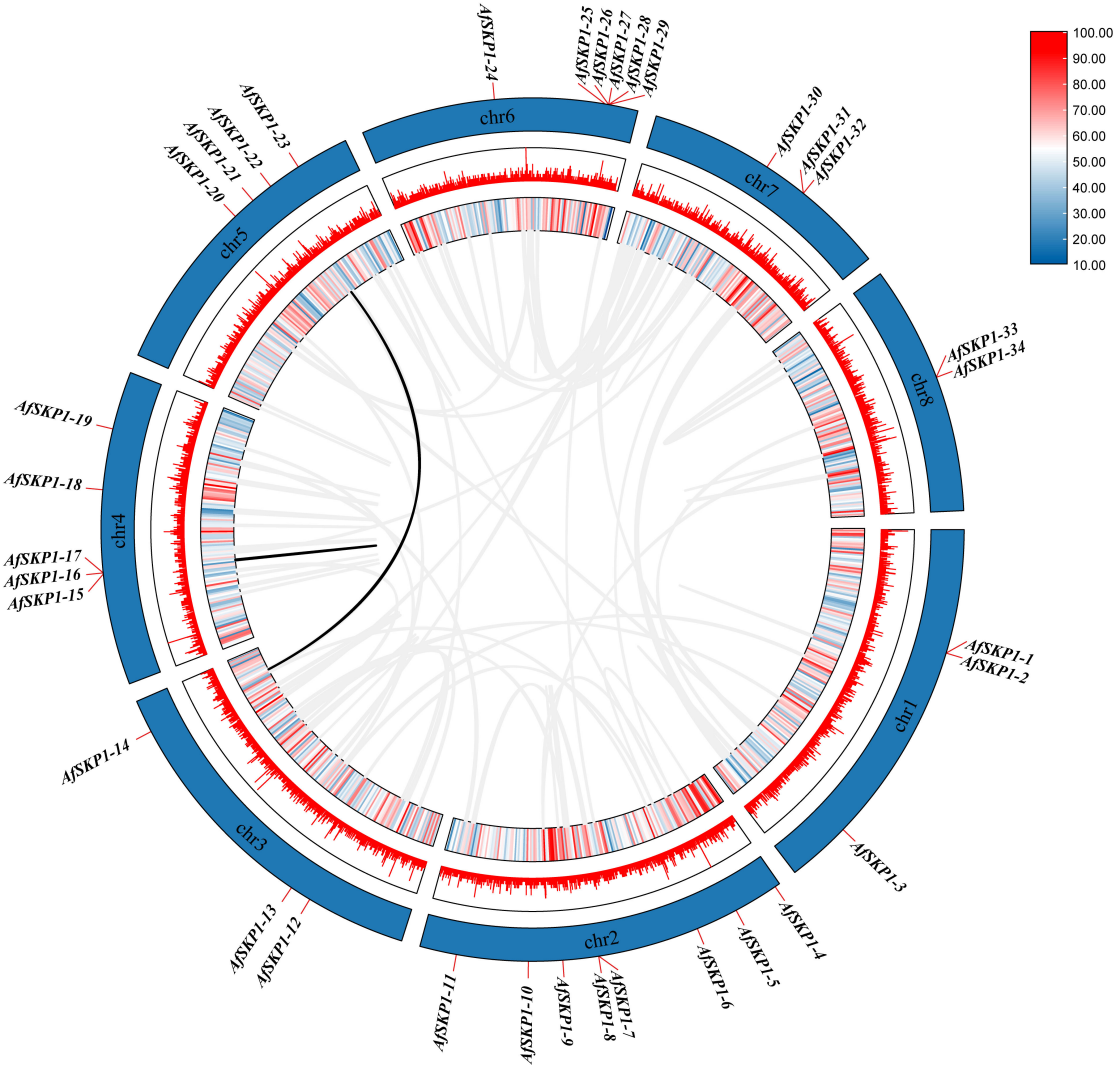
shows a schematic representation of the interchromosomal relationships and segmental duplication events of the *AfSKP1* genes. Gray lines represent all co-localized blocks in the *Allium fistulosum* genome, while black lines indicate duplicated *AfSKP1* gene pairs.

In this study, a collinearity map of *AfSKP1* genes was constructed (**Figure 7**), and the intraspecific collinearity of 34 members of the *AfSKP1* gene family was analyzed. The results revealed two homologous relationships among *AfSKP1* genes, along with collinearity relationships among their members, specifically *AfSKP1-22* and *AfSKP1-14*, *AfSKP1-15* and *AfSKP1-16*. These relationships were characterized by fragment replication. The evidence presented above indicates that gene replication has occurred in the *AfSKP1* gene family, suggesting that the *AfSKP1* gene may have expanded the family through replication during evolution.

### Analysis of promoter cis-acting elements of *AfSKP1* gene family members

**Figure 8:**
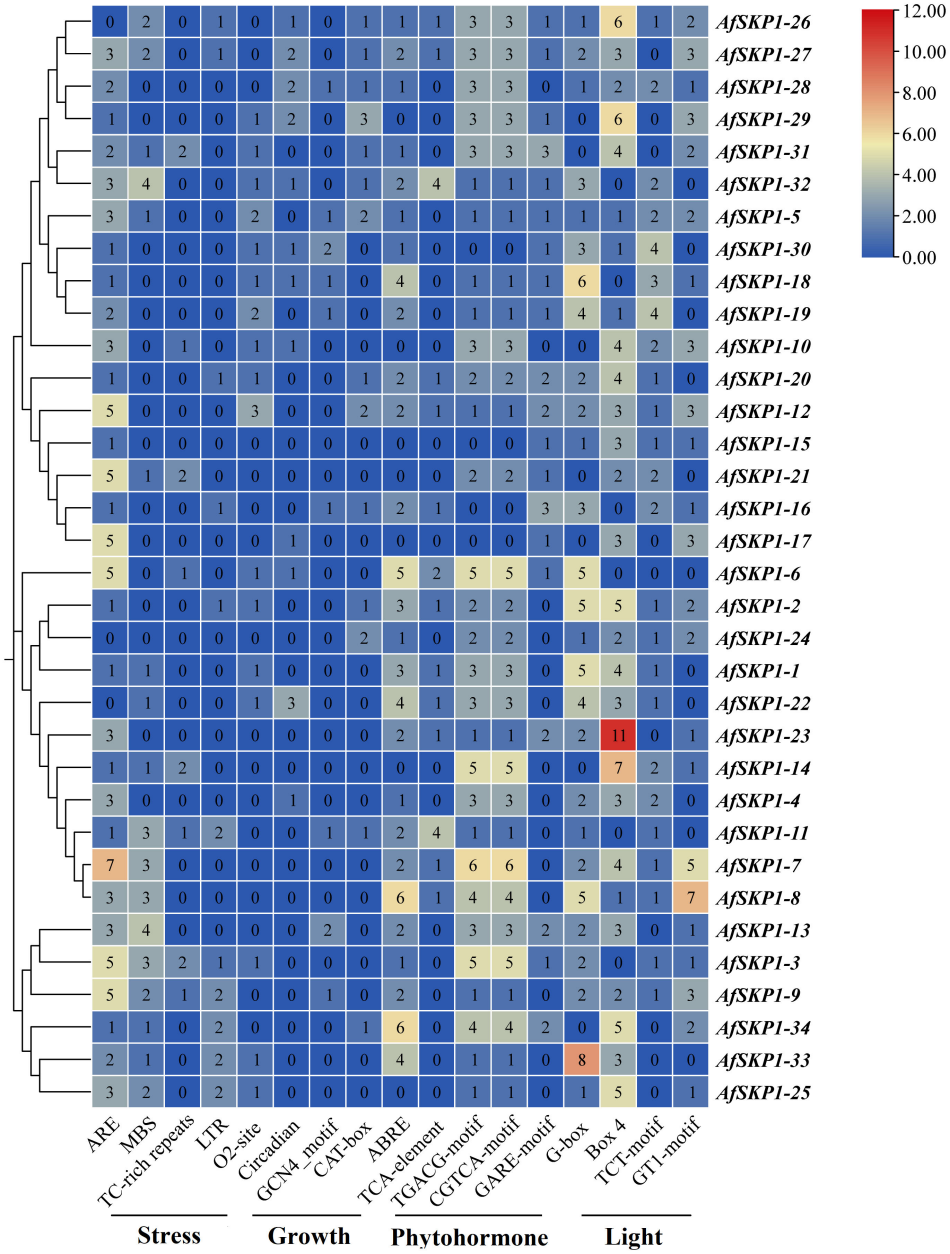
Cis-element distribution of*AfSKP1* genes. Blue, yellow, red, indicating the number of cis-acting elements from low to high, and the number in the heat map represents the number of cis-acting elements.

The cis-acting elements of the *AfSKP1* gene family members were analyzed. According to their functions, they can be divided into phytohormone response elements, light response elements, stress response elements, and plant growth and development elements (**Figure 8**). Among them, the number of cis-acting elements related to hormone response is the largest, totaling 269, including abscisic acid response element (ABRE), methyl jasmonate response element (CGTCA-motif, TGACG-motif), and salicylic acid response element (TCA-element), abscisic acid response element (GARE-motif). Methyl jasmonate can effectively induce flowering and inhibit flower bud differentiation, with the highest number of 91 response elements (Cao et al., 2016). Abscisic acid could induce the defense response of onion plants, showing strong disease resistance, with the second-highest number of 65 response elements. Salicylic acid could induce cell differentiation, with the lowest number of 29 response elements (Hu et al., 2022). This suggests that multiple hormones may be involved in regulating the expression of *AfSKP1*, jointly promoting *A. fisulosum* growth and flower development. The total number of light-responsive elements (Box 4, G-box, GT1 motif, and TCT motif) was 268, with Box 4 having the highest number (101), G-box the second highest (76). The *AfSKP1-23* gene accounted for the highest number of Box 4 elements, followed by *AfSKP1-14*. Box 4 is part of the DNA module involved in the light response, suggesting that the main regulatory features of these genes are light-related. Meanwhile, *AfSKP1-8* and *AfSKP1-23* had the highest number of light-responsive elements, indicating that these genes are closely related to light response. Among the stress response elements, ARE, which determines the binding of regulatory proteins and anaerobically induced expression, had the highest number of response elements, totaling 83. ARE elements were detected in all 31 genes, suggesting that the *AfSKP1* gene may be closely related to the anaerobic response of *A. fisulosum*. The total number of drought-associated MBS elements ranked second with 36, of which 17 promoters were able to detect MBS elements. Low-temperature response (LTR) elements totaled 16, and defense and stress response (TC-rich repeats) elements totaled 12. Additionally, some elements related to plant growth and development response were found, totaling 68, among which the promoter elements related to zeaxanthin metabolism (O2 site) were the most numerous, with a total of 21, suggesting their role in regulating *A. fisulosum* growth and development. Other cis-acting elements upstream of eukaryotic structural genes (CAT box), endosperm expression (GCN4 motif), and circadian-related cis-acting elements were also identified.

### Analysis of *AfSKP1* gene family expression patterns

**Figure 9:**
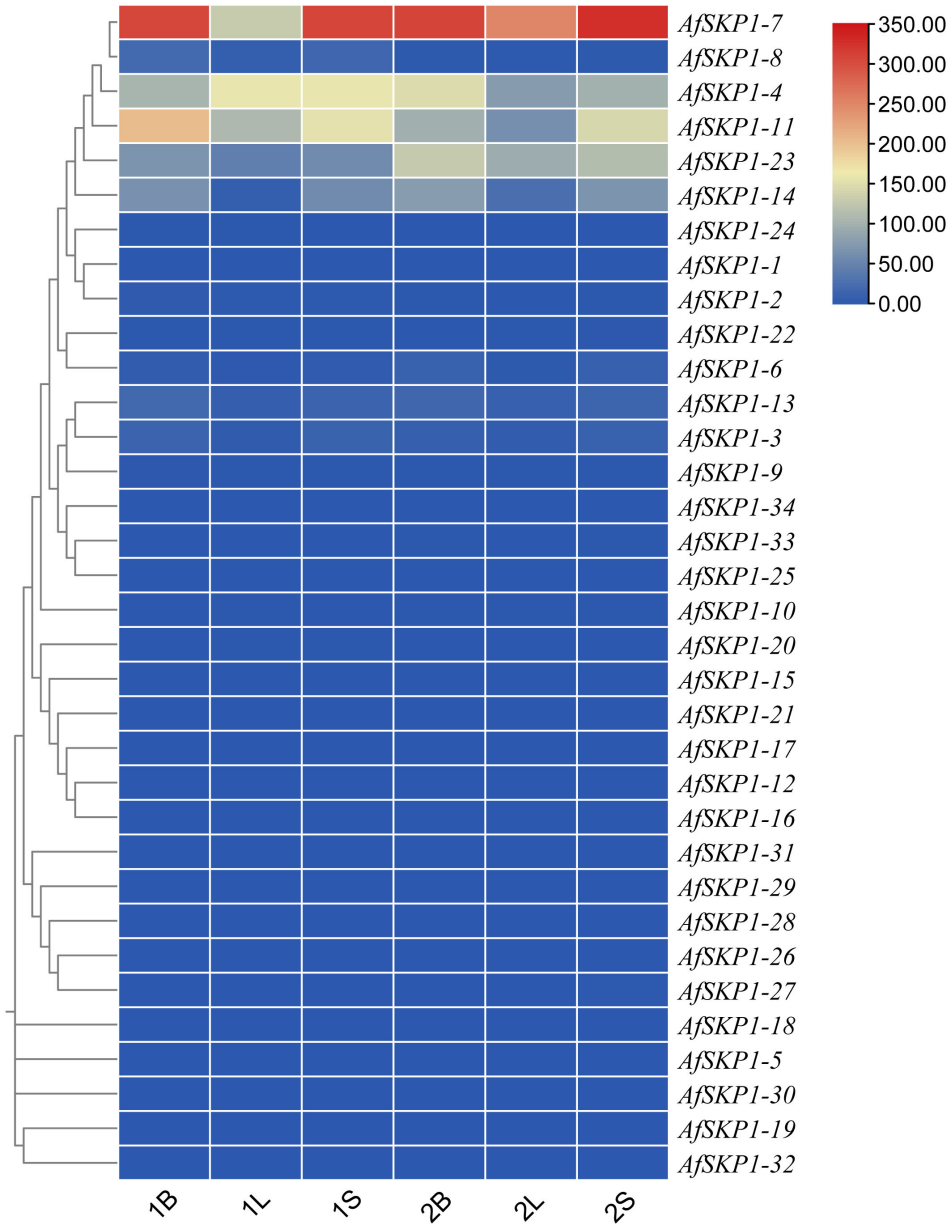
The heatmap of *AfSKP1* gene family members using the transcriptional datasets. Dark blue, light blue, yellow, light red and dark red are used to represent gene expression levels. Blue indicates weak gene expression, red indicates strong gene expression. 1 is *Allium fisulosum* var. *viviparum* Makinot, 2 is *Allium galanthum* B, L and S are floral buds, leaf and inflorescence meristems.

In order to explore the potential biological function of *AfSKP1* gene in the growth and development of *A. fistulosum*, the expression of *AfSKP1* gene in different tissues was analyzed by RNA-seq data. As shown in **Figure 9**, the expression levels of different genes in flower buds, leaves and flower buds were different, and the expression levels of 28 *AfSKP1* genes in different tissues of the two materials were generally low. The expression levels of *AfSKP1-4* in the inflorescence meristems of *A. galanthum* was higher than that in the inflorescence meristems of *A. fisulosum* var. *viviparum* Makinot, and the expression levels in the floral buds and leaves of *A. galanthum* were lower than those in the floral buds and leaves of *A. fisulosum* var. *viviparum* Makinot, respectively. *AfSKP1-7* was highly expressed in inflorescence meristems and floral buds of the two materials. It can be observed that the expression of *AfSKP1-11* in inflorescence meristems and floral buds of the two materials was higher than that in leaves. The expression levels of *AfSKP1-14* and *AfSKP1-23* in the inflorescence meristems and floral buds of *A. galanthum* surpassed those in the corresponding tissues of *A. fisulosum* var. *viviparum Makinot*, with both species displaying higher expression levels in inflorescence meristems and floral buds compared to leaves. The expression levels of *AfSKP1-11* and *AfSKP1-14* in the floral organs of both species at various developmental stages may contribute to the failure of anther dehiscence and pollen maturation. Furthermore, *AfSKP1-5*, *AfSKP1-15*, *AfSKP1-16*, *AfSKP1-18*, *AfSKP1-19*, *AfSKP1-21*, *AfSKP1-25*, *AfSKP1-26*, *AfSKP1-28*, *AfSKP1-29*, *AfSKP1-30*, *AfSKP1-31*, *AfSKP1-32*, and *AfSKP1-34* were not expressed in either species. These findings suggest that the AfSKP1 gene exhibits significant tissue-specific expression, indicating potential functional differentiation among the various *AfSKP1* members. In light of the previous identification of the differential gene *AfSKP1-8* and the expression analysis of *AfSKP1-11* and *AfSKP1-14*, this study will focus on these three genes for further investigation.

### Analysis of the *AfSKP1* protein interaction network

**Figure 10:**
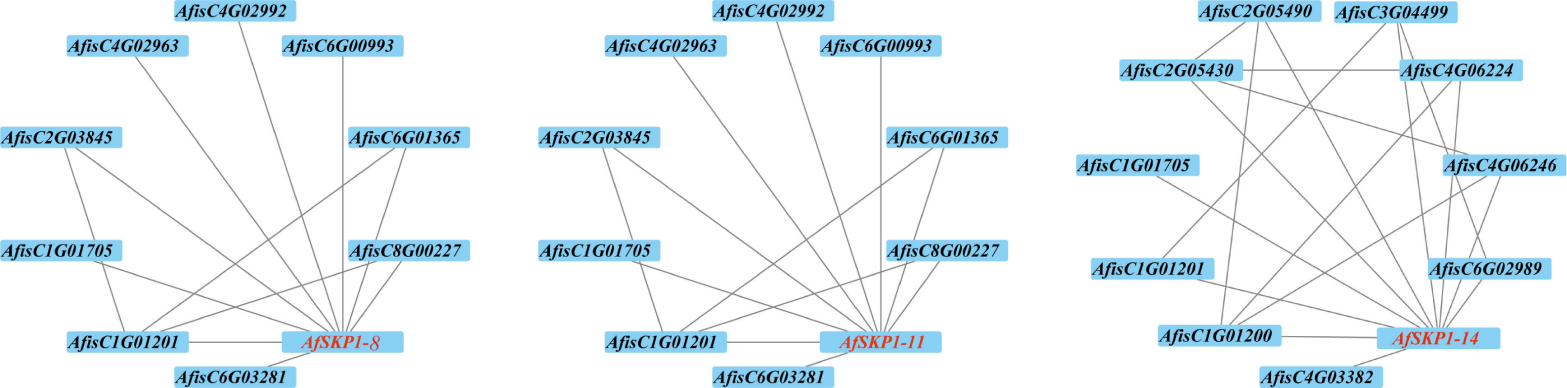
Inter-crossing network diagram of *AfSKP1-8, AfSKP1-11, AfSKP1-14*.

In order to better understand the function of the *AfSKP1* gene in *A.fistulosum*, this study predicted the interactions between *AfSKP1-8*, *AfSKP1-11*, *AfSKP1-14*, and other proteins based on the STRING online database. The results showed (**Figure 10**) that *AfisC4G01705* and *AfisC4G01201* both interact with these three proteins. *AfisC4G01705*, annotated as EIF3E, was confirmed to be involved in pollen germination and tube growth processes (Wang et al., 2008). *AfSKP1-7* and *AfSKP1-11* interacted with the same proteins, a total of 9 proteins, of which *AfisC4G02992* was annotated as *CEN2* and was confirmed to be involved in the regulation of the pollination process and cell elongation (He et al., 2022). This suggests that *AfSKP1* proteins may interact with *CEN2* and *EIF3E* proteins in the regulation of floral organ development and pollination. In addition, there are some proteins that are not annotated. They have clear direct or indirect synergistic effects with *AfSKP1* proteins, but their functions are not yet known.

### Expression profiles of *AfSKP1-8*,*AfSKP1-11*, and *AfSKP1-14* genes

**Figure 11:**
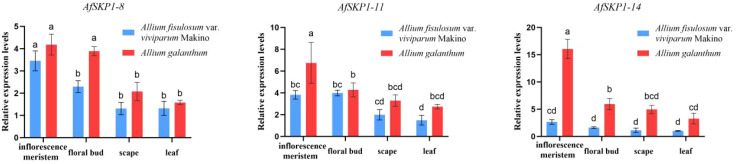
Expression of *AfSKP1-8, AfSKP1-11, AfSKP1-14* genes in different tissues. Different lowercase letters represent significant differences.

In this study, qRT-PCR was used to detect the transcription levels of the *AfSKP1* gene in inflorescence meristems, leaves, scapes, and floral buds of the two materials. The expression patterns of *AfSKP1-8*, *AfSKP1-11*, and *AfSKP1-14* genes in the two materials were verified. By analyzing the gene expression levels of the three *AfSKP1* gene families in different tissues and organs (**Figure 11**), it was found that the expression levels of these three *AfSKP1* genes varied across different tissues. There was no difference in the expression of the *AfSKP1-8* gene in inflorescence meristems, scapes, and leaves between the two materials. However, the expression level in floral buds was significantly different between the two materials (p < 0.05), with the expression level in *A.galanthum* being 1.69 times higher than that in *A.fistulosum* var. *viviparum* Makino. The expression level of the *AfSKP1-11* gene in floral buds, scapes, and leaves did not differ between the two materials, but the expression level in inflorescence meristems was significantly different (p < 0.05), with the expression level in *A.galanthum* being 1.76 times higher than that in *A.fistulosum* var. *viviparum* Makino. The expression of the *AfSKP1-14* gene in inflorescence meristems, scapes, and floral buds was significantly different between the two materials (p < 0.05). The expression level in inflorescence meristems of *A.galanthum* was 6.05 times higher than that of *A.fistulosum* var. *viviparum* Makino. In floral buds, the expression level in *A*.*galanthum* was 3.67 times higher than that in *A.fistulosum* var. *viviparum* Makino. Additionally, the expression level in scapes of *A.galanthum* was 4.37 times higher than that in *A.fistulosum* var. *viviparum* Makino. The genes that are highly expressed in floral organs may play a significant role in the floral development of the two studied materials. Notably, *AfSKP1-11* and *AfSKP1-14* exhibited significantly different expression levels in the floral buds of these materials (p < 0.05). This differential expression may contribute to the inability of the anthers of *A.fistulosum* var. *viviparum* Makino to undergo dehiscence, as well as the failure of pollen maturation. These findings suggest that the *AfSKP*1 gene family may be involved in the regulation of floral organ development in *A. fistulosum.* Furthermore, in conjunction with the results from paraffin section analysis, it is proposed that the genes *AfSKP1-8*, *AfSKP1-11*, and *AfSKP1-14* may be implicated in the failure of *A. fistulosum* var. *viviparum* Makino to produce mature pollen grains, ultimately resulting in pollen abortion.

## Discussion

Stigma fertility represents a critical component of sexual reproduction, with its fertile period significantly influencing the successful pollination and fertilization of flowers. The selection of male sterile materials necessitates that the stigma develops normally and is capable of facilitating effective pollination and fertilization. The material studied, *A.fisulosum* var. *viviparum* Makino, fulfills the criteria for male sterile material. Its remarkable drought and cold tolerance, coupled with strong environmental adaptability, offers valuable genetic resources for enhancing stress tolerance in other *Allium* species. The tapetum is essential for anther dehiscence and pollen release, as it provides both nutrients and space necessary for pollen development and release. Premature degradation of the tapetum impedes the nutrient supply required for pollen development, leading to delayed or aborted pollen development, which results in the failure of anthers to rupture. In the course of anther development, tapetal degradation and delayed programmed cell death contribute to male sterility in *Actinidia chinensis* Planch (Falasca et al., 2013). In model plants, many genes are implicated in these developmental processes. In the subsequent stage, as pollen grains differentiate and mature, structural changes in the anther wall occur, including the secondary thickening of the endothelial cell wall, which provides the mechanical force necessary for anther dehiscence (Nelson et al., 2012). This secondary wall thickening is typically achieved through the deposition of lignin; however, abnormal accumulation of lignin within the endothecium wall can disrupt normal anther dehiscence and pollen release (Thévenin et al., 2011). Consequently, if the anther fails to crack appropriately, the release of pollen is hindered, ultimately resulting in male sterility. The uneven thickening of the inner and outer walls of the fibroblasts determines the direction of anther dehiscence, while the V-shaped distribution of the secondary thickening of the fibroblasts on the anther surface determines the positional order of anther dehiscence (Zheng et al., 2019). The anther tapetum of *A.fisulosum* var. *viviparum* Makino degraded at the S12 stage, but it did not completely degrade, retaining a thin layer of cells. The volume of endothecium cells decreased, leading to the atrophy of the anther structure. Consequently, at the S13 stage, the anther chamber could not crack, and the pollen grains could not mature and release. This may be the main reason for the male sterility of the *A.fisulosum* var. *viviparum* Makino. Through transcriptome data analysis, we found that some members of the *SKP1* gene family were highly expressed at different stages of flower development. At present, the reported function of this gene family is relatively conserved and mainly acts on anther development. Therefore, it is speculated that it may also play a key role in male sterility of Allium plants.

S-Phase kinase-associated protein 1 (*SKP1*) is a small molecular weight protein commonly found in eukaryotes. Its primary biological function is to participate in the formation of the SCF complex, regulate ubiquitin-mediated protein degradation in organisms through the 26S protease pathway, and take part in various biological processes, including plant pollen development. The *SKP1* gene family has been extensively studied in many plants such as *A. thaliana, O. sativa, Hordeum vulgare, Triticum aestivum*, and *Lilium brownii* (Hoopen et al., 2000; Sasaki et al., 2003; Wang and Yang, 2006; Chang et al., 2009; Hong et al., 2013; Senda et al., 2013). However, there are limited reports on the *SKP1* gene family of *A.fistulosum*. Conducting a genome-wide analysis of its genes will enhance our understanding of the gene family. In this study, 34 *AfSKP1* genes were identified from the *A.fistulosum* genome. Gene structure analysis revealed that the number of introns in the *AfSKP1* genome ranged from 1 to 11, which was higher than that in crops such as almonds (Zhang et al., 2023). It is speculated that the frequency of recombination between *AfSKP1* genes may increase, which could be more conducive to the evolution of *A.fistulosum* and have corresponding regulatory effects.

The phylogenetic and evolutionary relationships of *SKP1* genes in four representative species (*A*. *fistulosum, A*. *thaliana, S*. *lycopersicum*, and *O*. *sativa*) were investigated through phylogenetic tree construction. The analysis divided them into three groups. *SKP1* genes within the same group exhibit a high degree of homology, suggesting that homologous genes within the group may have originated from a common ancestor through replication. These genes are believed to have similar functions. Gene replication is a key factor contributing to the rapid expansion and evolution of gene families (Jouffrey et al., 2021). The *AfSKP1* gene family shows evidence of gene replication, indicating that the *AfSKP1* gene may have been amplified through replication during evolution.

Cis-regulatory elements are crucial molecular switches involved in the transcriptional regulation of genes under abiotic or biotic stresses, playing a significant role in gene expression regulation (Nakashima et al., 2009; Wang et al., 2017). In this study, an analysis of cis-acting elements in the 2000 bp promoter region upstream of *AfSKP1* revealed a variety of elements related to plant growth and development, as well as cis-elements associated with abiotic stresses. These include elements involved in cell cycle regulation, hormone responsiveness, low-temperature response, and drought response. Additionally, most members of the *AfSKP1* family responded to multiple hormone regulations, such as ABA, MeJA, and other hormones involved in plant stress signaling, indicating the family’s importance in growth, development, and stress response. Specifically, most *AfSKP1* members responded to MeJA and ABA regulation. Hormones like ABA and MeJA can effectively induce flowering and inhibit flower bud differentiation. ABA exhibited a slight inhibitory effect on the expression of the *GhSKP1* gene in the leaves of Gossypium hirsutum (Hu et al., 2013). By analyzing the expression of the *AfSKP1* gene in various tissues, it becomes evident that the expression levels of different *AfSKP1* genes vary significantly across tissues. This suggests that the *AfSKP1* gene exhibits distinct tissue-specific expression, implying potential biological functional diversity among different *AfSKP1* members. Variances in gene expression levels were observed between inflorescence meristems and floral buds, leading to speculation that these genes may play crucial roles in the development of flower organs in *A.fistulosum*.

The expression of *AfSKP1* gene in different tissues was analyzed by RNA-seq data. The expression levels of different genes in Leaves, inflorescence meristems and floral buds were different, and the expression levels of 28 *AfSKP1* genes in different tissues of the two materials were generally low. The expression of *AfSKP1-11* in flower buds and flower buds was higher than that in leaves. The expression levels of AfSKP1-14 in inflorescence meristems and floral buds of *A. galanthum* were higher than those in inflorescence meristems and floral buds of *A.fistulosum* var. *viviparum* Makino, and the expression levels of the two materials in inflorescence meristems and floral buds were higher than those in leaves. Genes highly expressed in floral organs may be involved in the flower development of the two materials. Through qRT-PCR analysis, *AfSKP1-8, AfSKP1-11, AfSKP1-14* were expressed in different tissues, but also showed its tissue expression specificity. The differential expression of *AfSKP1-8*, *AfSKP1-11* and *AfSKP1-14* in inflorescence meristems and floral buds is consistent with the trend of RNA-seq data, and the expression level in flower organs is higher than that in other tissues. We speculate that the low expression of *AfSKP1-8*, *AfSKP1-11* and *AfSKP1-14* in *A.fistulosum* var. *viviparum* Makino may lead to the failure of *A.fistulosum* var. *viviparum* Makino to develop into mature pollen grains, resulting in the final abortion of pollen. In *G. hirsutum*, the high expression of *GhSKP1* in the radicle and stamen (the part containing dividing cells) suggests that *GhSKP1* may be involved in cotton growth and flower development (Hu et al., 2013). Overexpression of Paeonia suffruticosa Andrews *SKP1* increased the expression of flowering-related genes *CO* (Constans), *LFY* (LEAFY), and *FT* (Flowering Locus T), promoting flower formation and early flowering. The expression of *PSK1* (Paeonia Skp1-like) genes was progressively enhanced during the development of flower buds and the flowering process (Hao et al., 2017). We initially hypothesized that the *AfSKP1* gene may have functions involved in the growth and influence pollen development of *A. fistulosum* var. *viviparum* Makino.

## Conclusion

In this study, the *AfSKP1* gene family was screened and identified using bioinformatics methods. The structure and conserved domains of these genes were studied, and the characteristics of *AfSKP1* family members were analyzed from the perspectives of phylogenetic evolution, chromosome distribution, protein physicochemical properties, and motif prediction. Additionally, the promoter cis-acting elements, expression patterns, and protein interaction networks of gene family members were analyzed. In addition, it was also found that the tapetum of the onion was not completely degraded, resulting in the anther chamber could not crack, and the pollen grains could not mature and release. At the same time, the expression patterns of *AfSKP1* in different tissues and organs were analyzed based on RNA-seq and qRT-PCR., revealing its potential involvement in anther development and pollen grain maturation of *A.fistulosum* var. *viviparum* Makino, ultimately leading to male sterility. It was further confirmed that A*fSKP1-8*, *AfSKP1-11*, and *AfSKP1-14* may be candidate genes involved in anther development, providing a theoretical foundation for further research on *AfSKP1* family members related to floral organ development and pollen development. Moreover, it was discovered that *A.fistulosum* var. *viviparum* Makino could serve as male sterile material for breeding purposes.

## Data Availability

The data presented in the study are deposited in the NCBI repository, accession number PRJNA1139370

## References

[B1] Bailey T. L. Boden M. Buske F. A. Frith M. Grant C. E. Clementi L.. (2009). MEME SUITE: tools for motif discovery and searching. Nucleic Acids Res. 37, W202–W208. doi: 10.1093/nar/gkp335 19458158 PMC2703892

[B2] Cao J. J. Li M. Y. Chen J. Liu P. Li Z. (2016). Effects of MeJA on Arabidopsis metabolome under endogenous JA deficiency. Sci. Rep. 1, 37674. doi: 10.1038/srep37674 PMC5121592 27883040

[B3] Chang L. Guo C. L. Lin Y. S. Fu H. Wang C. S. Jauh G. Y. (2009). Pollen-specific *SKP1-like* proteins are components of functional scf complexes and essential for lily pollen tube elongation. Plant&Cell. Physiol. 8, 1558–1572. doi: 10.1093/pcp/pcp100 19578169

[B4] Chen C. Chen H. Zhang Y. Thomas H. R. Frank M. H. He Y.. (2020). TBtools: an integrative toolkit developed for interactive analyses of big biological data. Mol. Plant 8, 1191–1202. doi: 10.1016/j.molp.2020.06.009 32585190

[B5] Dafni A. (1992). Pollination Ecology (New York: Oxford University Press), 1–57.

[B6] Ewing B. Hillier L. Wendl M. C. Green P. (1998). Base-calling of automated sequencer traces using phred. I. Accuracy assessment. Genome Res. 3, 175–185. doi: 10.1101/gr.8.3.175 9521921

[B7] Falasca G. D’Angeli S. Biasi R. Fattorini L. Matteucci M. Canini A.. (2013). Tapetum and middle layer control male fertility in *Actinidia deliciosa* . Ann. Bot. 6, 1045–1055. doi: 10.1093/aob/mct173 PMC3783237 23965617

[B8] Francesca L. Uwe B. Apoorva B. (2016). Different promoter affinities account for specificity in MYC-dependent gene regulation. Elife 5, 15161. doi: 10.7554/eLife.15161 PMC4963202 27460974

[B9] Gasteiger E. Gattiker A. Hoogland C. lvanyi l. Appel R. D. Bairoch A. (2003). ExPASy: The proteomics server for in-depth protein knowledge and analysis. Nucleic Acids Res. 13, 3784–3788. doi: 10.1093/nar/gkg563 PMC168970 12824418

[B10] Hao Q. Ren H. X. Zhu J. Wang L. S. Huang S. C. Liu Z. A.. (2017). verexpression of PSK1, a SKP1-like gene homologue, from Paeonia suffruticosa, confers salinity tolerance in Arabidopsis. Plant Cell Reports. 1, 151–162. doi: 10.1007/s00299-016-2066-z 27787596

[B11] He Y. Li L. Y. Shi W. B. Tan J. H. Luo X. X. Zheng S. Y.. (2022). Florigen repression complexes involving rice CENTRORADIALIS2 regulate grain size. Plant Physiol. 2, 1260–1274. doi: 10.1093/PLPHYS/KIAC338 PMC9516737 35861433

[B12] Hellmann H. Estelle M. (2002). Plant development: regulation by protein degradation. Science 5582, 793–797. doi: 10.1126/science.1072831 12161644

[B13] Hong M. J. Kim D. Y. Seo Y. W. (2013). SKP1-like-related genes interact with various F-box proteins and may form SCF complexes with Cullin-F-box proteins in wheat. Mol. Biol. Rep. 2, 969–981. doi: 10.1007/s11033-012-2139-1 23065282

[B14] Hoopen R. Manteuffel R. Dolezel J. Malysheva L. Schubert I. (2000). Evolutionary conservation of kinetochore protein sequences in plants. Chromosoma 7, 482–489. doi: 10.1007/s004120000109 11151678

[B15] Hu Y. L. Zhi L. L. Li P. Hancock J. Hu X. Y. (2022). The role of salicylic acid signal in plant growth, development and abiotic stress. Phyton-International. J. Exp. Bot. 12, 2591–2605. doi: 10.32604/PHYTON.2022.023733

[B16] Hu D. L Chen Q. Z Zhang C. J. Wang Y. Zhang B. Z. Tang C. M. (2013). Identification of cotton SKP1-like gene GhSKP1 and its function in seed germination and taproot growth in tobacco. Canadian Journal of Plant Science 93(5), 817–825. doi: 10.4141/CJPS2012-312

[B17] Jouffrey V. Leonard A. S. Ahnert S. E. (2021). Gene duplication and subsequent diversifification strongly affect phenotypic evolvability and robustness. R. Soc. Open Sci. 6, 201636–201636. doi: 10.1098/RSOS.201636 PMC8220273 34168886

[B18] Kim Y. Y. Jung W. K. Jeung U. J. Shin J. ,. S. (2011). A novel F-box protein represses endothecial secondary wall thickening for anther dehiscence in *Arabidopsis thaliana* . J. Plant Physiol. 2, 212–216. doi: 10.1016/j.jplph.2011.09.006 22018967

[B19] Kim D. Langmead B. Salzberg S. L. (2015). HISAT: a fast spliced aligner with low memory requirements. Nat. Methods 4, 357–360. doi: 10.1038/nmeth.3317 PMC4655817 25751142

[B20] Krzywinski M. Schein J. Birol I. Connors J. Gascoyne R. Horsman D.. (2009). Circos: an information aesthetic for comparative genomics. Genome Res. 9, 1639–1645. doi: 10.1101/gr.092759.109 PMC2752132 19541911

[B21] Kumar S. Steche G. Li M. Knyaz C. Tamura K. (2018). MEGA X: molecular evolutionary genetics analysis across computing platforms. Mol. Biol. Evol. 6, 1547–1579. doi: 10.1093/molbev/msy096 PMC5967553 29722887

[B22] Lescot M. Déhais P. Thijs G. Marchal K. Moreau Y. Peer Y. V.. (2002). PlantCARE, a database of plant cis-acting regulatory elements and a portal to tools for in silico analysis of promoter sequences. Nucleic Acids Res. 1, 325–327. doi: 10.1093/nar/30.1.325 PMC99092 11752327

[B23] Li Y. Jiang J. Du M. L. Li L. W. Xiu L. Li X. B. (2013). A cotton gene encoding MYB-like transcription factor is specifically expressed in pollen and is involved in regulation of late anther/pollen development. Plant Cell Physiol. 6, 893–906. doi: 10.1093/pcp/pct038 23447105

[B24] Liu A. L. Yu Y. Duan X. B. Sun X. L. Duanmu H. Z. Zhu Y. M. (2015). *GsSKP21*, a Glycine soja S-phase kinase-associated protein, mediates the regulation of plant alkaline tolerance and ABA sensitivity. Plant Mol. Biol. 2, 111–124. doi: 10.1007/s11103-014-0264-z 25477077

[B25] Love M. I. Huber W. Anders S. (2014). Moderated estimation of fold change and dispersion for RNA-seq data with DESeq2. Genome Biol. 15, pp. doi: 10.1186/s13059-014-0550-8 PMC4302049 25516281

[B26] Michael G. (2021). All about *allium* . Curr. Biol. 22, R1449–R1452. doi: 10.1016/j.cub.2021.11.006

[B27] Mitsuda N. Iwase A. Yamamoto H. Yoshida M. Sek M. Shinozaki K.. (2007). NAC transcription factors, NST1 and NST3, are key regulators of the formation of secondary walls in woody tissues of Arabidopsis. Plant Cell. 1, 270–280. doi: 10.1105/TPC.106.047043 PMC1820955 17237351

[B28] Nakashima K. Yusuke I. Kazuko Y. (2009). Transcriptional regulatory networks in response to abiotic stresses in Arabidopsis and grasses. Plant physiology. 149, 88–95. doi: 10.1104/pp.108.129791 19126699 PMC2613698

[B29] Nelson M. R. Band L. R. Dyson R. J. Lessinnes T. Wells D. M. Yang C.. (2012). A biomechanical model of anther opening reveals the roles of dehydration and secondary thickening. New Phytol. 4, 1030–1037. doi: 10.1111/j.1469-8137.2012.04329.x PMC3569878 22998410

[B30] Pertea M. Pertea G. M. Antonescu C. M. Tsung C. Mendell J. T. Salzberg S. L. (2015). StringTie enables improved reconstruction of a transcriptome from RNA-seq reads. Nat. Biotechnol. 3, 290–295. doi: 10.1038/nbt.3122 PMC4643835 25690850

[B31] Prakash A. Jeffryes M. Bateman A. Finn R. D. (2017). The HMMER web server for protein sequence similarity search. Curr. Protoc. Bioinf. 1, 3.15.1–3.15.23. doi: 10.1002/cpbi.40 29220076

[B32] Rose A. B. (2018). Introns as gene regulators: A brick on the accelerator. Front. Genet. 672. doi: 10.3389/fgene.2018.00672 PMC6374622 30792737

[B33] Sanders P. Q. Bui A. Q. Weterings K. (1999). Anther developmental defects in Arabidopsis thaliana male-sterile mutants. Plant Reproduction. 11, 297–322. doi: 10.1007/s004970050158

[B34] Sasaki A. Itoh H. Miyako G. Kanako U. T. Kanako I. Kobayashi M.. (2003). Accumulation of phosphorylated repressor for gibberellin signaling in an *F-box* mutant. Science 5614, 1896–1898. doi: 10.1126/science.1081077 12649483

[B35] Senda K. Imen B. E. H. Aurélia B. Ferchichi A. Kong H. Z. Mouzeyar S.. (2013). Structural, expression and interaction analysis of rice *SKP1-like* genes. DNA Res. 1, 67–78. doi: 10.1093/dnares/dss034 PMC3576659 23248203

[B36] Su G. Morris J. H. Demchak B. Bader G. D. (2014). Biological network exploration with cytoscape 3. Curr. Protoc. Bioinf. 1, 8.13.1–8.1324. doi: 10.1002/0471250953.bi0813s47 PMC4174321 25199793

[B37] Sundaresan V. Springer P. Volpe T. Haward S. Jones J. Dean C.. (1995). Patterns of gene action in plant development revealed by enhancer trapand gene traptransposable elements. Genes&Development 9, 1797–1810. doi: 10.1101/gad.9.14.1797 7622040

[B38] Szklarczyk D. Gable A. L. Lyon D. Junge A. Wyder S. Huerta-Cepas J.. (2018). STRING v11: protein–protein association networks with increased coverage, supporting functional discovery in genome-wide experimental datasets. Nucleic Acids Res. D1, D607–D613. doi: 10.1093/nar/gky1131 PMC6323986 30476243

[B39] Thévenin J. Pollet B. Letarnec B. Saulnier L. Gissot L. Maia-Grondard A.. (2011). The simultaneous repression of CCR and CAD, two enzymes of the ligninbiosynthetic pathway, results in sterility and dwarfism in arabidopsis thaliana. Mol. Plant 1, 70–82. doi: 10.1093/mp/ssq045 20829305

[B40] Trapnell C. Williams B. A. Pertea G. Mortazavi A. (2010). Transcript assembly and quantification by RNA Seq reveals unannotated transcripts and isoform switching during cell differentiation. Nat. Biotechnol. 5, 511–515. doi: 10.1038/nbt.1621 PMC3146043 20436464

[B41] Wang W. Jiang W. Liu J. G. Li Y. Gai J. Y. Li Y. (2017). Genome-wide characterization of the aldehyde dehydrogenase gene superfamily in soybean and its potential role in drought stress response. BMC Genomics 18, 518. doi: 10.1186/s12864-017-3908-y 28687067 PMC5501352

[B42] Wang M. Y. Song Y. L. Zhang S. X. Zhao X. L. Wang J. W. Niu N.. (2015). The analysis of *SKP1* gene expression in physiological male sterility induced by chemical hybridizing agent SQ-1 in wheat (*Triticum aestivum* L.). Cereal Res. Commun. 2, 204–212. doi: 10.1556/crc.43.2015.2.3

[B43] Wang Y. Tang H. Debarry J. D. Tan X. Li J. Wang X.. (2012). MCScanX: a toolkit for detection and evolutionary analysis of gene synteny and collinearity. Nucleic Acids Res. 7, e49. doi: 10.1093/nar/gkr1293 PMC3326336 22217600

[B44] Wang Y. X. Yang M. (2006). The Arabidopsis *SKP1-LIKE1* (ASK1) protein acts predominately from leptotene to pachytene and represses homologous recombination in male meiosis. Planta 3, 613–617. doi: 10.1007/S00425-005-0154-3 16283376

[B45] Wang Y. Zhang W. Z. Song L. F. (2008). Transcriptome analyses show changes in gene expression to accompany pollen germination and tube growth in Arabidopsis. Plant Physiol. 3, 1201–1211. doi: 10.1104/pp.108.126375 PMC2577266 18775970

[B46] Wu Q. S. Bazzini A. A. (2023). Translation and mRNA stability control. Annu. Rev. Biochem. 92, 227–245. doi: 10.1146/ANNUREV-BIOCHEM-052621-091808 37001134

[B47] Xu C. Park S. J. Van E. J. Lippman Z. B. (2016). Control of inflorescence architecture in tomato by BTB/POZ transcriptional regulators. Genes Dev. 18, 2048–2061. doi: 10.1101/gad.288415.116 PMC5066612 27798848

[B48] Xu Y. F. Tanushri S. Lokesh K. Minella A. C. (2010). MicroRNA-223 regulates cyclin E activity by modulating expression of *F-box* and WD-40 domain protein 7. J. Biol. Chem. 45, 34439–34446. doi: 10.1074/jbc.M110.152306 PMC2966058 20826802

[B49] Yamashita K. Iino M. Shigyo M. Tashiro Y. (2000). Visualization of Nucleus Substitution between *Allium galanthum* and Shallot (*A. cepa*) by Genomic *In Situ* Hybridization. J. Japanese. Soc. Hortic. Sci. 2, 189–191. doi: 10.2503/jjshs.69.189

[B50] Yang M. Hu Y. Lodhi M. McCombie W. R. Ma H. (1999). The Arabidopsis *SKP1-LIKE1* gene is essential for male meiosis and may control homologue separation. Proc. Natl. Acad. Sci. 96, 11416–11421. doi: 10.1073/pnas.96.20.11416 10500191 PMC18048

[B51] Yang C. Y. Song J. Ferguson A. C. Klisch D. Simpson K. Mo R.. (2017). Transcription factor MYB26 is key to spatial specificity in anther secondary thickening formation. Plant Physiol. 1, 333–350. doi: 10.1104/pp.17.00719 PMC5580765 28724622

[B52] Yang P. Yuan Y. Yan C. Jia Y. You Q. Da L. L.. (2024). AlliumDB: a central portal for comparative and functional genomics in Allium. Horticult. Res. 2, 285–285. doi: 10.1093/HR/UHAD285 PMC10871970 38371639

[B53] Yao Q. Bai Y. Kumar S. Au E. Orfao A. Chim C. S. (2020). Minimal residual disease detection by next-generation sequencing in multiple myeloma: A omparison with real-time quantitative PCR. Front. Oncol. 10. doi: 10.3389/fonc.2020.611021 PMC7878533 33585233

[B54] Zhang D. D. Yu Z. F. Hu S. B. Liu X. Y. Zeng B. Gao W. W.. (2023). Genome-wide identification of members of the *Skp1* family in almond (*Prunus dulcis*), cloning and expression characterization of PsdSSK1. Physiol. Mol. Biol. Plants. 1, 35–49. doi: 10.1007/s12298-023-01278-9 PMC9886703 36733834

[B55] Zhao D. Z. Ni W. M. Feng B. M. Han T. Petrasek M. G. Ma H. (2003). Members of the Arabidopsis-SKP1-like gene family exhibit a variety of expression patterns and may play diverse roles in Arabidopsis. Plant Physiol. 1, 203–217. doi: 10.1104/pp.103.024703 PMC196598 12970487

[B56] Zheng L. J. Nagpal P. Villarino G. (2019). miR167 limits anther growth to potentiate anther dehiscence. Dev. (Cambridge. England). 14, dev174375–dev174375. doi: 10.1242/dev.174375 31262724

