# Supplementary figures and images for: Transcriptome and genome-wide analysis of the potential role of SKP1 gene family in the development of floral organs of two related species of Allium fistulosum
Source: Front Plant Sci. 2024 Nov 7;15:1470780. doi: 10.3389/fpls.2024.1470780 (PMC11578749; doi:10.3389/fpls.2024.1470780)

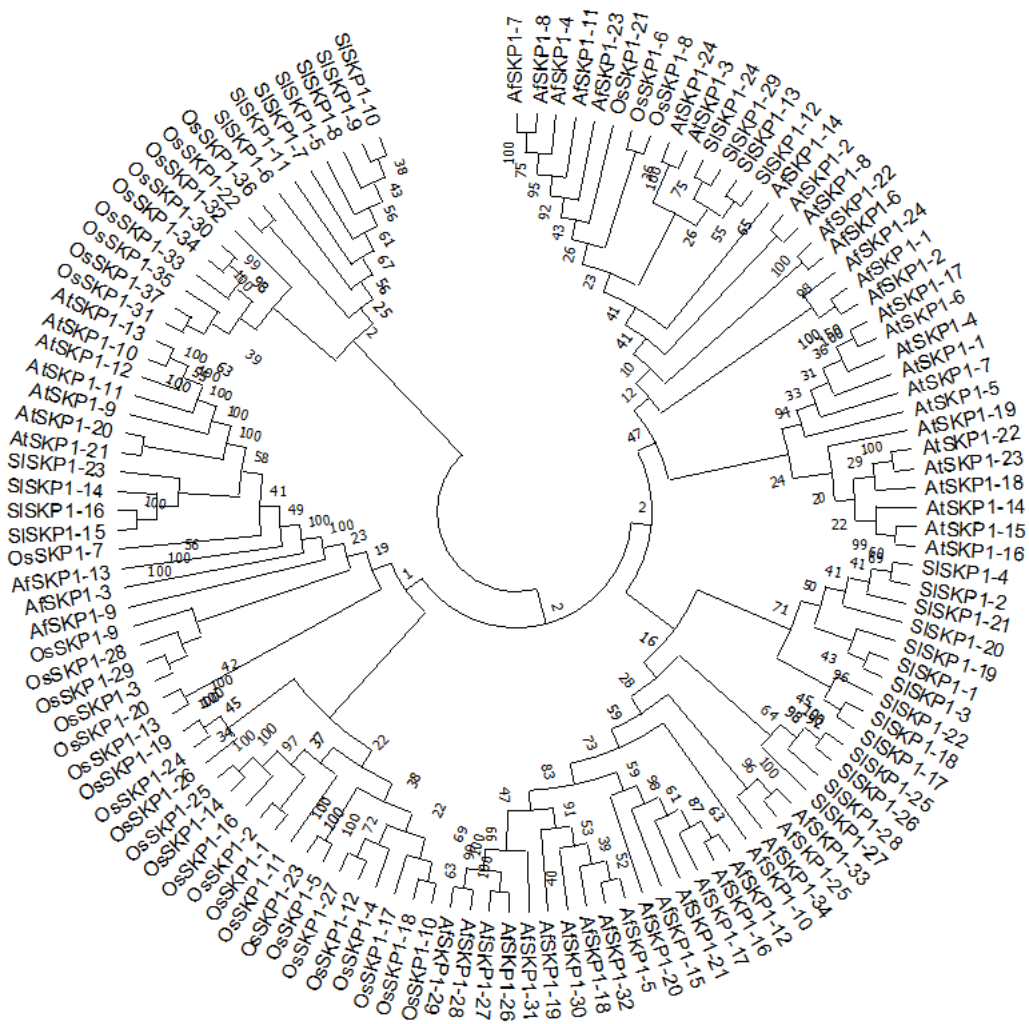

Supplement: Supplementary file 1 [file DataSheet1.pdf]
